# Supplementary material for: Characterizing the evolution and phenotypic impact of ampliconic Y chromosome regions
Source: Nat Commun. 2023 Jul 6;14:3990. doi: 10.1038/s41467-023-39644-6 (PMC10326017; doi:10.1038/s41467-023-39644-6)
Supplement: Supplementary file 1 — Supplementary Information [file 41467_2023_39644_MOESM1_ESM.pdf]

**SUPPLEMENTARY METHODS**

I- Copy number estimations

**1) Nomenclature**

We classified amplicon CN genotypes using a system of nomenclature devised to describe recurrent deletions and duplications in the AZFc region of the Y chromosome (reviewed in [1], Figure 1F). This nomenclature provides information about the location of deletion or duplication breakpoints, by listing the first letter of the amplicon colour label (g for green, r for red, etc. because it was originally detected with coloured probes using FISH.) and the numerical order of amplicon instances along the Y chromosome reference sequence (r1 and r4 for the first and fourth instances of the red amplicon). Thus, for example, *gr/gr* deletions result from a pairing of *r1*, *r2* and *g1* with *r3*, *r4* and *g3* followed by a recombination event that leads to a deletion of *b3*, *y1*, *g2*, *r3* and *r4* (Figure 1F).

**2) Dataset**

We estimated copy number of 11,527 males from Iceland, part of Decode sequencing dataset, including 7,947 males belonging to 1,449 patrilines.

All individuals were sequenced using PCR-free method at a mean coverage of 30X.

Haplogroup assignment was performed using HaploGrouper [2] and the 2016 version of the ISOGG Y chromosome phylogenetic tree.

**3) Construction of the artificial Y chromosome**

We first constructed a reduced Y chromosome fasta sequence composed of the proximal arm sequences for palindrome 1, 3, 4, 5, 6, 7 and 8 of the Y chromosome, in which all annotated genes were masked. The Y-linked single copy genes and one copy of each ampliconic gene sequence (+- 2kb upstream and downstream) were then added to the final Y chromosome mapping file (see Supplementary Table 1, 2, 3 for the hg38 positions of the sequences included).

**4) Mapping, filtering and get the coverage**

The pipeline from [3] and [4] was used for determining the copy number of genes. To reduce computation time, instead of using the fastq files for mapping to the artificial Y chromosome, we used the bam files already mapped to the reference Y chromosome. We extracted the reads that mapped to the Y chromosome and remapped them to the artificial Y chromosome and the X chromosome to ensure that, for gametologs, reads mapping preferably to X homologs would map to the X. The median coverage of all Y single copy genes was used as a control.

*BWA* 0.7.5 [5] was used to perform the mapping (mem -M -a). *sambamba* 0.5.1 [6] was used to filter the paired reads, sort the reads per coordinates, remove the duplicates and filter the bam files for a mapping quality >= 50. We allowed for a number of mismatches lower or equal to 1 base pair and used the cigar option =˜/151M/. After mapping to the artificial chromosome, the coverage for each position was obtained using *SAMtools* 1.3 [7].

The median coverages of all single copy genes were used to obtain a measure of the expected coverage for single copy for each individual. Copy number of regions of interest can then be estimated

by dividing the median coverage of the regions of interest by the expected coverage for single copy. Single copy genes are AMELY, DBY, EIF1AY, KDM5D, NLGN4Y, PCDH11Y, PRKY, RPS4Y1, RPS4Y2, SRY, TBL1Y, TGIF2LY, TMSB4Y, TXLNGY, USP9Y, UTY and ZFY. Individuals with a median coverage for all single copy genes lower than 6 reads were removed.

Following a reviewer's comment, we evaluated if removing two genes from our set of single copy genes changes the CN estimates. Specifically, we removed TGIF2LY and PCDH11Y, which have a very similar homolog on the X chromosome that could lead to reads mis-mapping. Although, we used the X chromosome in the initial mapping so reads should map preferentially there.

Excluding TGIF2LY and PCDH11Y decreases the mean coverage of single copy genes over the 11,525 males from 12.41 to 12.25. However, since we are using the median of the median read depth of all 17 single copy genes to standardize the median read depth across positions for our CN estimation of the target regions, the inclusion of these two genes has a minimal impact on our CN estimation. Both median read depths over the 11,525 males, including or excluding TGIF2LY and PCDH11Y, is equal to 12.

We estimated copy number for the yellow amplicon, using the single copy genes coverage without TGIF2LY and PCDH11Y. Supplementary Figure 14 illustrates the differences between both CN estimates (using the median coverage). Only 7 individuals out of 11,525 show a difference in CN higher than 0.4 copy. For one individual, the CN estimation increases by one copy (from six to seven copies). For the others, the CN is classified into ambiguous copy number. None of these changes modified our patriline mutation rate.

### 5) Estimating copy number

Palindromes 1, 2 and 3 contains distinct ampliconic elements that are found in different number of copies along the Y chromosome. These amplicons are termed yellow, green, red, blue and teal (see Figure 1A), and were considered separately. For palindrome 1 (P1), 2 (P2) and 3 (P3), copy number was assessed using the coverage from the amplicons blue, teal, yellow, green and red. P1 copy number was estimated from the yellow region, which is unique to P1, and P3 copy number was estimated from the teal region, which is unique to P3. In the reference Y chromosome, the red region is present in 2 copies in P1 and 2 copies in P2, the green region is present in 1 copy next to P2 and in two copies in P1, the blue region is present in 2 copies in P1 and 2 copies in P3 (Figure 1A). Reads belonging to the blue regions all mapped on P3. P2 was not included in the artificial Y chromosome but copy number could be estimated using the red and green region, mapping on P1. For palindrome 4 to 8, copy number was estimated using the coverage of the proximal palindromic arm.

#### *a. Using the median coverage over the whole region*

For each individual and each region of interest, we divided the median coverage of the palindromic arms or amplicons by the median coverage of all single copy genes. For the amplicon yellow, green and teal, and for palindromes 4 and 5, we computed the median coverage over several windows (Supplementary Table 20). The coverage of all the windows was considered in the calculation of the

median coverage of the region. The positions of the windows were chosen to avoid low coverage regions, notably due to the masking of the genes located in palindromic arms in the artificial chromosome.

*b. Using the HMM*

We also inferred copy number along the amplicons and palindromes using a hidden Markov model with copy numbers as hidden states. The HMM assigns copy number based on the median coverage of non-overlapping sliding windows and uses the median coverage of all single copy gene as a reference for the 'one copy' state. The probability of staying in the same state was set to 0.999, and the distribution of the HMM was a Poisson distribution, with a parameter defined as the median coverage of all single copy genes. For the amplicons located in P1, P2 and P3, windows of 5000 bp were used in the HMM, while windows of 1000 bp were used for the other palindromes because they are much smaller (Supplementary Table 1, 2, 3). Compared to the first method, it gives an integer estimate, allows for changes in copy number within a region and can detect duplication and deletion breakpoints. Windows with more than 10% of missing data were not taken into account. For yellow, green and teal amplicons and P4 and P5, several sub-regions were removed from the HMM analyses due to low coverage, because the genes were masked in the reference sequence or because of poor mapping (Supplementary Table 20).

*c. Using the median coverage over windows of 1000bp*

We also estimated the copy number of amplicons and palindromes over non-overlapping windows of 1kb. For each window, the median coverage was computed and divided by the median coverage of single copy genes. The median of the median coverage of each window was then computed.

**6) Filtering for ambiguous copy number**

Differences between copy number inference from the HMM and the median coverage methods can either indicate uncertainty in the estimation or a change in copy number along the palindrome or amplicon considered. We therefore identified individuals with either *i)* a breakpoint in copy number within an amplicon, detected by the HMM or *ii)* individuals without evidence of a change in copy number along the sequence but with a difference between estimates of copy number from the median and from the HMM of more than half of a copy (Supplementary Table 4).

*a. Several windows in the HMM*

We evaluated the validity of the breakpoint assessment by the HMM. For each individual with a breakpoint, the median CN was calculated for sliding windows of 1000bp over the region considered (see section I.5.c). The other individuals, *i.e.* individuals that do not have breakpoints detected by the HMM and for which the difference between median and HMM estimations is less than 0.5, were used as a reference set and grouped in clusters of CN defined by the HMM estimates.

For each window, we compared the median CN of the focal individual with the median CN of all the individuals located in the cluster of CN above and below the HMM calculation. For example, if the

breakpoint in CN is from 2 to 3 copies, the median CN of each window was compared with individuals with a CN of 2 and 3, independently. The difference between the median of the individual and the median of the medians of all individuals of both clusters was recorded for each window. We therefore had two measurements of differences per window: the difference of the medians with the cluster up (in the example, CN of 3) and the cluster down (in the example, CN of 2). For each window of 1000bp, we then assessed which of the difference, up or down, was the lowest.

We then considered the positions of the segments discovered by the HMM, and calculated how many times the cluster up was closer than the cluster down in this segment, and divide it by the number of windows in this segment. Therefore, each segment is assigned one value: the ratio of the number of times the cluster up was closer to the median CN of the individual over the cluster down in this segment. For example: if the HMM discovered that from position 1000 to 23000, the CN was 2 and from position 23000 to 80000, the CN was 3, the ratio was calculated over the windows included in 1000-23000 and over the windows included in 23000-80000. If the cluster up is in majority the closest over a given segment, the value of the ratio will be high, and *vice-versa*. For each individual, if all the segments have a ratio value < 0.25 or > 0.75, we consider that the change of CN within the amplicon is real (Supplementary Figure 15). Otherwise, the CN is categorized as ambiguous, and a new CN is defined as 'CN down-CN up' (for example, 2-3). This analysis was not performed in cases where the cluster up or down was composed of less than 5 individuals, for such cases the CN was also categorized as ambiguous and defined as 'CN down-CN up'. For palindromes 4 to 8, individuals with a breakpoint within the palindrome was automatically assigned to an ambiguous CN, because the number of individuals with a CN different from the reference is low.

For the green amplicon, we could confirm 65 out of the 70 breakpoints tested (Supplementary Figure 15). These individuals are counted in different categories in Supplementary Table 6 depending on the other amplicon CN (4 in P1+P2, 55 in P2 and 4 in the breakpoint green category).

For the yellow amplicon, we could confirm 4 breakpoints within yellow using the above pipeline (Supplementary Figure 15). Moreover, we detected 29 individuals with a partial complete deletion of yellow, that were assigned to CN 0. The above pipeline could not be applied as there is no "down" cluster. However, the partial deletion was confirmed visually (Supplementary Figure 4) and is unlikely to be an artefact as all individuals have the same haplogroup and 25 of them belong to two patrilines (N=23 and N=2). They are counted in Supplementary Table 6 as the 29 "P1" events.

*b. Discrepancy between the HMM and the median*

Individuals with a difference of copy number estimates between the HMM and the median of the medians over 1kb windows (>=0.5) were assigned to an ambiguous copy number: the median rounded down to the closest integer and the median rounded up to the closest integer, that contains the HMM state (for example 2-3, which means 2 or 3). For P4 to P8, only P7 showed individuals with discrepancy (81). Individuals showing discrepancy do not seem to be enriched for individuals showing a high variance within the CN calculated by window (Supplementary Figure 16), but they are consistently located at the edges of CN clusters (Supplementary Figure 17). This indicates that individuals located near the boundary of a CN cluster are more likely to have false assignment of copy number.

*c. Applying a cutoff for the edge of the clusters*

As shown above, individuals located at the vicinity of CN cluster edges have CN estimates less trustworthy. Thus, we defined a zone around integer values where the CN of individuals is categorized as non ambiguous. Values falling outside of this interval were defined as ambiguous.

For each of the clusters of CN with more than 5 individuals, we applied the following:

We used the median of the medians, calculated in non-overlapping windows of 1kb (see section I.5.c) and the median CN calculated over the whole region (see section I.5.a). If, for an individual, the median of the medians or the median calculated over the whole region is not located within the boundary of confidence of a cluster (CN of the cluster +- 0.35), then its copy number is defined as ambiguous « median rounded down to the closest integer - median rounded up to the closest integer ». If the CN cluster had 5 individuals or less only (except for 0 copy), we applied an ambiguous CN.

The individuals that were assigned an ambiguous copy number because they showed discrepancy of CN between the HMM and the median coverage estimates (section I.6.b) were also assigned an ambiguous CN by this cut-off.

*d. Categorizing individuals with ambiguous CN*

Individuals were categorized has having an ambiguous CN if they belonged in one of 3 categories: *i)* breakpoint detected by the HMM but not validated, *ii)* difference >=0.5 between the HMM and the median of the medians per window CN estimates and *iii)* located outside of their cluster CN +- 0.35 (Supplementary Figure 17). They were not removed from further analyses but their ambiguity in CN was considered for the detection of events in patrilines and in the Y chromosome tree.

**7) Assessing the accuracy of the CN estimates using duplicates**

The availability of individuals which have been sequenced independently more than once (duplicates) allowed us to investigate the robustness of our copy number estimations. Two sets of duplicated sequencing were available: from different sample types (blood and buccal, 77 individuals) and from the same sample type (both blood or both buccal but from different runs of sequencing, 14 individuals). The same pipeline to assess CN was applied to the duplicates, and the CN estimates were compared for each pair of duplicates. Individuals with ambiguous CN were not considered for this analysis.

We report the differences in CN estimated using the median coverage over the whole amplicon (section 1.5.a, Supplementary Table 23) and using the HMM (section 1.5.b, Supplementary Table 24). For duplicates with the same sample type, no differences were detected from the HMM estimates, and the CN estimated by the median are very similar between duplicates. We observe larger differences between duplicates from different sample types overall. Amplicon green and red, as well as palindrome 7, show differences in CN estimated with the HMM (Supplementary Table 24, false positive rate of 0.013, 0.026 and 0.024, respectively).

Overall, this analysis indicates that our CN estimates can be trusted for amplicon yellow and teal, and P4, P5, P6 and P8. For the other regions, while the false positive rate is low, caution has to be

applied when assessing a mutational event.

**8) Determining the palindromes copy number from the amplicons**

An in-house python code was used to determine the copy number of the palindromes from the copy number of the amplicons. Copy number for palindrome 1 is the copy number of yellow, copy number for palindrome 2 is the copy number of red divided by two and copy number for palindrome 3 is the copy number of teal. We then determined if individuals have a different copy number than the reference for one or several palindromes.

For individuals with an ambiguous CN, if one of the possible CN was the reference CN, we counted it as reference. For individuals with breakpoints, we determined if the other amplicons also showed a mutation: if yes, the mutation was classified as such (for example, if green had 3.5 copies and red had 5 copies, we classified it in 'P2').

**9) Validation methods based on independent mapping**

For a subset of 9162 individuals, mapped independently to the entire reference genome (hg38) [8] two different validation methods were performed.

*a. Allelic Balance Analysis*

We used lastz (Harris *et al.* 2010), to locate paralogous sequences in the yellow amplicon and combined all reads per paralogous position per individual. Genotype calling was then performed on these combined reads. Given the high sequence identity between amplicons, most of the combined genotypes do not display any variation and are referred to as *pseudo*-homozygous since they represent alleles on a haploid chromosome, not alleles on diploid sister chromosomes. Occasionally there is variation, or *pseudo*-heterozygosity, meaning that one of those paralogous positions carries a mutation, hereafter referred to as paralogous sequence variant (PSV). Allelic frequencies were calculated on these PSVs. Calculated allelic frequencies at PSVs can inform us about the copy number, as there is only a subset of values possible for each copy number.

We used PSVs, where at least one male is pseudo-heterozygous. A number of filters were implemented per PSV in order to exclude false positives.

The minor allele read frequency (MARF) at PSVs has an expectation of min(n0,n1) / N, where N is the number of yellow amplicon copies carried by a male and n0 and n1 are the number of copies with the ancestral (0) and derived (1) allele, respectively. PSVs failing on any of these filters were excluded from further analysis:

A- PSVs must be polymorphic

B- PSVs must have one paralogous position

C- the minor allele frequency per position must be over 0.1

D- PSVs displaying more than three types of genotypes per position were excluded (for example, if the possible alleles are A and G, we allow for genotype AG, G and A)

E- each PSV must have more than 5 reads mapping

An in-house script was used to extract and summarize all pseudo-heterozygous genotypes per male per PSV. Two new filters were then applied:

1- genotypes involving indels were excluded

2- a minimum of two possible alleles must be present in pseudo-heterozygous genotypes

These filters result in 4139 PSVs for palindrome 1 and 2334 polymorphic positions for the yellow amplicon.

Allele frequencies were calculated per PSV per male using an in-house script. Then, the average allele frequency was computed per male for the yellow amplicon.

*b. Relative sequence depth*

We performed a second validation analysis using relative sequence depth (rdepth) as a proxy for CN.

Samtools bedcov was implemented to determine the average sequence depth per individual BAM file for the X-degenerate regions, a total of 8,974,361 positions (see Supplementary Table 25 for the exact coordinates used). The average sequence depth for all individuals included was 19.51x (sd=7.26) and the median was 18.8x. We used lastz [9] to obtain a list of paralogous positions in the yellow amplicon and used them to calculate, for each male, the per-position depth for yellow 1 and yellow 2 combined. The relative depth (rdepth) for each position in each male was obtained using as a denominator the average per-position sequence depth from the X-degenerate regions. Our new CN estimate was then based on the average of means or medians of rdepth in 1000bp windows across the yellow amplicon.

A comparison of these CN estimates with those estimated as part of the main analysis reveals a mismatch rate of 0.0003 for the median and 0.001 for the mean, where a mismatch was recorded when our independent estimate of rdepth differed by more than 0.5 from our original estimate. Rdepth CN estimates were calculated both including and excluding individuals categorized as 0 CN owing to most of them carrying a partial deletion of the yellow amplicon (Supplementary Methods section I.6.a, Supplementary Figure 4), revealed by their median and mean values being more in line with either 2 CN and 1 CN (see Supplementary Table 9).

### 10) Classification of known events

Individuals with an ambiguous CN comprising the reference CN were assigned to the reference group. Individuals with a CN different from the reference were classified into different classes of known deletion using diagnostic copy number of amplicons (Table 1). For the other individuals, the type of event as classified as 'unknown'.

### 11) Clustering of the events

A PCA was performed using the R package FactoMineR [10]. A density-based clustering on the principal component of the PCA was computed using the R package dbscan [11]. An additional cluster was added for the one individual carrying the b1/b3 deletion (Supplementary Figure 7, cluster 13). Cluster 3 encompasses 2 types of known duplications, and was therefore divided into cluster 3 (b2/b3 or g1/g3 duplication) and cluster 14 (gr/gr duplication). From the previously categorized unknown events (228 individuals), 212 individuals could be classified in one cluster while 16 did not belong to

any cluster.

II- Mutation rate within patrilines

### 1) Filtering of the patrilines

The patrilines were corrected using SNP genotype data from the X-degenerate region of the Y chromosome (as described in [12]). For each patriline, the number of genotype differences in the X-degenerate regions between each pair of males was evaluated against the threshold E + Nm, where E=5 is the maximum number of differences due to errors, N=2 is the maximum number per generation due to mutations and m is the number of generations separating the males in the patriline. Pairs with differences lower than the threshold were retained in the same patriline, whereas males who exceeded the threshold in all pairs were excluded from the analysis.

### 2) Algorithm for detecting events and assessing ancestral state

We used a parsimony algorithm, as described in Cunningham et al. (1998), to infer genotype states for ungenotyped ancestors in the patriline trees and thereby to assign mutation events in cases where genotype variation was observed among sequenced patriline members

### 3) Mutation rate estimates for MD and SD events

To assess the ancestral state of a patriline, if it was not possible using the tree, we defined the ancestral state as the reference CN if one of the CN states was the reference. Otherwise, we checked which was the most probable ancestral state using the tree and the distribution of CN in the haplogroup of the individuals. If all else failed, we assigned an unknown CN as ancestral state, and the direction of the event could not be determined.

Each event was categorized as Multiple Descendent (MD) if the mutation had several carriers or Single Descendent (SD) if the mutation had one carrier. It was further determined whether an event was an amplification or a deletion, if the ancestral state had been determined.

The events were manually curated for individuals that were assigned an ambiguous CN. If an individual with an ambiguous CN in one of the amplicon was carrier of an SD event in another amplicon and if the ambiguity of the CN in this individual removed the event, we would remove it from further analyses. For example, we removed an event found only in the green amplicon (3->4) because the individual has an ambiguous CN in red (4-5) and yellow (2-3), and the ambiguity of the CN in the copy number removes the SD event in green. On the contrary, if an event in the green amplicon is 3->6 and the ambiguity in yellow and red is 2-3 and 4-5, the events is still counted because the amplitude of the event is larger than the ambiguity in CN.

The counts of MD, SD, amplification, deletion and unknown events was performed for each amplicon and palindrome. This curated set of events was then regrouped into independent sets of events occurring in several or one amplicon only (the event involved yellow, green and red, for example).

### 4) Validation samples

We analysed additional individuals from two different datasets: the individuals that were sequenced using non-PCR-free methods and that were therefore removed from this analysis, and the individuals that have been newly sequenced since the beginning of this project. From these two complementary datasets, we selected individuals that are direct relatives (son, grand-son, father or grand-father) of individuals identified as bearing a mutation within a patriline.

Therefore, we were able to test four events by using extra individuals that were not included in our analysis and that are grand-sons, sons or fathers of an individual bearing a mutation.

Four individuals were added from the set of newly sequenced individual since the project started, including two from the same patriline that are the son and grand-son of the individual with an event (SD in green) and two from different patrilines (two MD in green, yellow and red). One individual was added from the set sequenced with non-PCR-free method (SD in green, red and yellow).

The four events tested, including two MD events and two SD events, were validated by the extra individuals (Supplementary Figure 19).

### 5) Mutation probability matrix

We calculated the mutation probability of each CN state by dividing the matrix of amplification/deletion of the *de novo* events by the number of individuals carrying this CN in the sample considered. We assumed that the patrilines represent a random sample of the population, and used the proportions seen in the whole sample. We removed states 0 copy and 4 copy, from which we did not have any events from and would therefore artificially create equilibrium at 0 and 4 copies. For other cases where we did not find any events, we applied a low mutation probability (0.0001). The R library markovchain [13] was used to infer the steady state of the proportions of the different CN in a population.

### 6) Rescue events analysis

The counts of the PSVs per individual per genomic regions are indicated in Supplementary Table 14. For the yellow amplicon, individuals annotated as ´2 copies, no event´ have between 53 and 57 PSVs, while individuals annotated as ‘2 copies, duplication’ have 4 PSVs, which is the same value as individuals with one copy. While the sequence depth is concordant with two copies in those individuals, the count of PSVs is concordant with individual bearing one copy, indicating that a unique copy was recently duplicated and that the event from one to two copies is real.

## III- <u>Phylogenetic mutation rate in the Y chromosome phylogenetic tree</u>

### 1) Definition of a set of individuals to build the tree

To construct a phylogenetic tree based on the Y chromosomes of the Icelandic males used in study, we first identified a set of good quality polymorphic SNP variants from the X-degenerate regions (8,974,361 positions). Specifically, we called genotypes for all X-degenerate positions using the approach described in [12] and sought SNPs that were variable among the set of 11,527 males analysed in this study. The SNPs selected (N=39,001) were those where all alleles were supported by >5 reads in total and >3 reads on average and where 99% of the males were assigned unambiguous haploid genotypes.

In order to minimize the overlap between CN mutation events detected in the phylogenetic tree and the patrilines and to make the construction of the phylogenetic tree more tractable, we pruned the full set of males down to a subset, such that no pair of males was separated by fewer than 10 genotype differences at SNPs. This led to the removal of 10,636 males, leaving 891 males to be used for constructing the phylogenetic tree. We used the neighbour-joining algorithm, as implemented in RapidNJ [14](https://birc.au.dk/software/rapidnj), to construct the tree from pseudo-sequences derived from the haploid genotypes of the 891 males at 23,118 variable SNPs. To obtain branch length estimates, the SNPs were assigned to the branches of the neighbour-joining tree (using a parsimony algorithm) and then the SNP mutation rate for the X-degenerate regions [12] was used to calibrate the branch lengths to meiosis, yielding a total of 83,909 meiosis in the tree (95% CI 75,765.9 - 93,333.5, based on the SNP mutation rate confidence intervals) and using 34.1 years per meiosis as a conversion factor to years. The branch structure of the neighbour-joining tree was then used to detect CN mutation events.

**2) Algorithm for detecting changes in CN within the tree**

The same parsimony algorithm used for the patrilines was used for the phylogenetic tree. The ancestral state of the tree is the reference CN.

**3) Phylogenetic mutation rate estimations**

The phylogenetic mutation rate was estimated by dividing the number of events by the number of meiosis included in the tree. The number of meiosis in the phylogenetic tree is 108639.05. Phylogenetic mutation rates were calculated for each amplicon. The confidence intervals were defined using a proportion test. Events were also grouped into independent set of events to obtain the phylogenetic mutation rate in the AZFc region (Supplementary Table 17).

IV- <u>Phenotypic associations</u>

**1) Test of the fertility phenotypes**

First, we considered two phenotypes that are proxies of fertility: the proportion of individuals with no children and the mean number of children. We compared these values between different groups of individuals with a CN mutation and the reference, using a Fisher exact test and a Wilcoxon Mann Whitney test, respectively. We applied two filters on birth year, first for individuals born before 1980 and second for individuals born before 1970. Categories including less than 15 individuals were not tested.

**2) Other phenotypes**

A curated set of 342 quantitative phenotypes was used to test association with CNV.

We compared non-reference CN with reference CN, deletion with reference CN and duplication with reference CN. Logistic regressions were performed for binary phenotypes, and linear regression were performed for quantitative phenotypes.

## 3) Simulations

We performed simulations to determine the level of selection (parameter s) acting on non-reference CN that would be necessary to explain the observed frequencies of non-reference CN in Iceland, considering the mutation matrix.

We first simulated a source population of 5,000 males with the reference CN (2 copies) for the yellow amplicon, evolving under the matrix of mutation probabilities constructed using the *de novo* mutation matrix (Supplementary Table 16). We performed 100 repetitions of 500 generations. Each generation were coded as follow:

**1-** One male is drawn from the population, each male has a probability to be drawn of 1-s/N, s being determined by his CN (reference or non-reference)

**2-** His number of children is drawn from a Poisson distribution of parameter 1

**3-** His son(s) CN are determined using the matrix of mutation probabilities

**4-** Back to 1- until there are 5,000 sons

The parameter s was simulated for values comprised between 0.0 and 0.03, with a step of 0.002 from 0.0 to 0.02 and then a step of 0.01 from 0.02 to 0.03. We assume that both amplification (>2 copies) and deletion (<2 copies) are equally deleterious. The simulations were performed using in-house python scripts, available upon request.

An overview of the result is presented in Supplementary Figures 11 and 12. The selection coefficients that lead to the closest proportions of individuals for each CN compared to what is observed in Iceland are s=0.01, s=0.012 and s=0.014 (Supplementary Figures 11 and 12, Supplementary Table 26).

## 4) Power analyses

We performed power analyses of a Student test (t test) between two mean numbers of children.

$N_0$: number of individuals with reference CN

$N_1$: number of individuals with non-ref CN

s: selection coefficient

We draw $N_0$ times a number of children from a Poisson distribution with a lambda of 2.44, which is the observed mean number of children in individuals bearing the reference CN, and $N_1$ times a number children from a Poisson distribution with a lambda of 2.44-(2.44)*s. Then, we perform a Student t test between those two distributions of number of children. We repeat this process 1000 times and calculate the power, which corresponds to the percentage of test that detects a difference in mean number of children out of the 1000 repetitions (two-sided Student t test, p-value <0.05).

First, we varied $N_0$ and $N_1$, assuming that both sets keep the same proportions than in the Icelandic population (0.914 for $N_0$ and 0.086 for $N_1$) and s is equal to 0.018 (Supplementary Figure 13A). For a sample size of 12,000 individuals, the power to detect a difference in mean number of children for s=0.018 is 16.0%.

Second, we varied the s from 0.0 to 0.2 with a step of 0.005 and fixed the sample size to the observed sample size in our analysis ($N_0$=10460, $N_1$=927, Supplementary Table 19). For an s of 0.018, the power is 16.3% (Supplementary Figure 13B). For a sample size corresponding to the number of

individuals with *de novo* mutations ($N_0$=7178, $N_1$=31), for s=0.018, the power is 5.9% (Supplementary Figure 13C).

The power simulations were performed using in-house R scripts, available upon request.

## SUPPLEMENTARY FIGURES

**Supplementary Figure 1: Flowchart of the method to estimate copy number. A- Construction of the reference sequence**, including the proximal arm of each palindrome with the genes masked, the single copy genes and the ampliconic genes (not shown) sequences. **B- Mapping of the reads of one individual on the reference sequence**, and computation of the coverage per position for each region considered. The black lines represent a simplification of the coverage distribution over the reference sequence, in the case where the individual has the reference copy number for each region. The median coverage of the single copy genes for this individual is used as a control. Here, we take an example with the single copy genes having a median coverage of 15 reads per position. Reads for amplicon blue map to palindrome 3 proximal arm. **C- Determination of copy number from the coverage**. The median coverage computed per 1kb window is represented (example from real data). Copy number (CN) per 1kb windows is estimated by dividing the median coverage of the window by the median coverage of single copy genes. **D-** We applied two methods to assess CN, both using the coverage of the region of interest and comparing it to the coverage of the single copy genes: **1- from median coverage** of the region of interest, computed using the median of the medians of the coverage over 1kb windows, represented in green; **2- from an HMM**, which determines an integer CN, represented in red. Amplicon yellow, green and red are taken into account separately, to account for their presence in several palindromes.

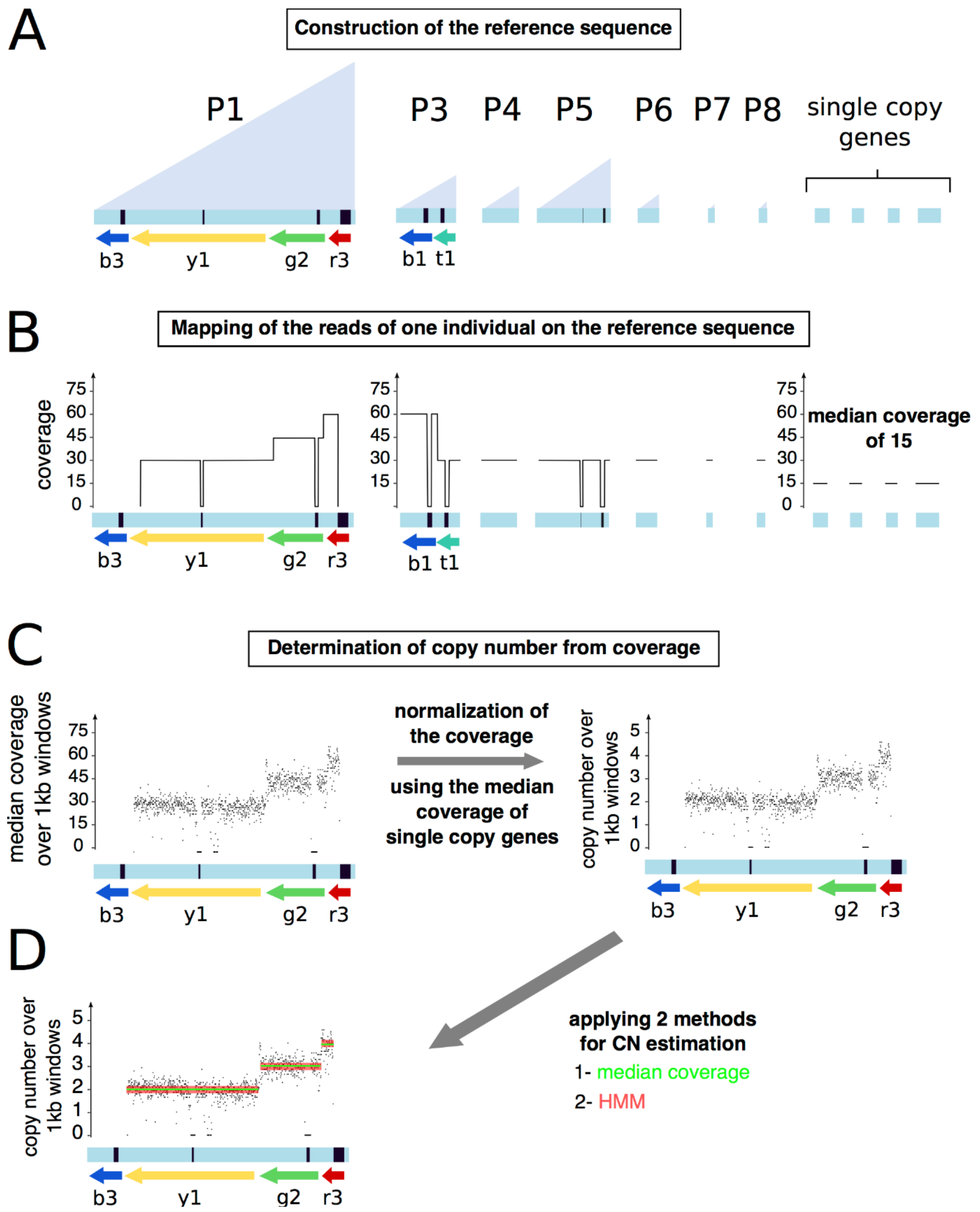
A
Construction of the reference sequence
P1
P3
P4
P5
P6
P7
P8
single copy genes
b3
y1
g2
r3
b1
t1
B
Mapping of the reads of one individual on the reference sequence
coverage
75
60
45
30
15
0
median coverage of 15
b3
y1
g2
r3
b1
t1
C
Determination of copy number from coverage
median coverage over 1kb windows
normalization of the coverage
using the median coverage of single copy genes
copy number over 1kb windows
D
copy number over 1kb windows
applying 2 methods for CN estimation
1- median coverage
2- HMM

**Supplementary Figure 2: Copy number for amplicons yellow, green, red, teal and blue** in palindrome 1, 2 and 3 in each haplogroup of the Icelandic population, estimated by the median of the median coverages over 1kb windows divided by the median coverage of single copy genes. Each dot represents an individual and is coloured according the copy number estimated using the HMM. Individuals with ambiguous copy number are not represented. Haplogroup I1a2 has experienced more dramatic changes in copy number.

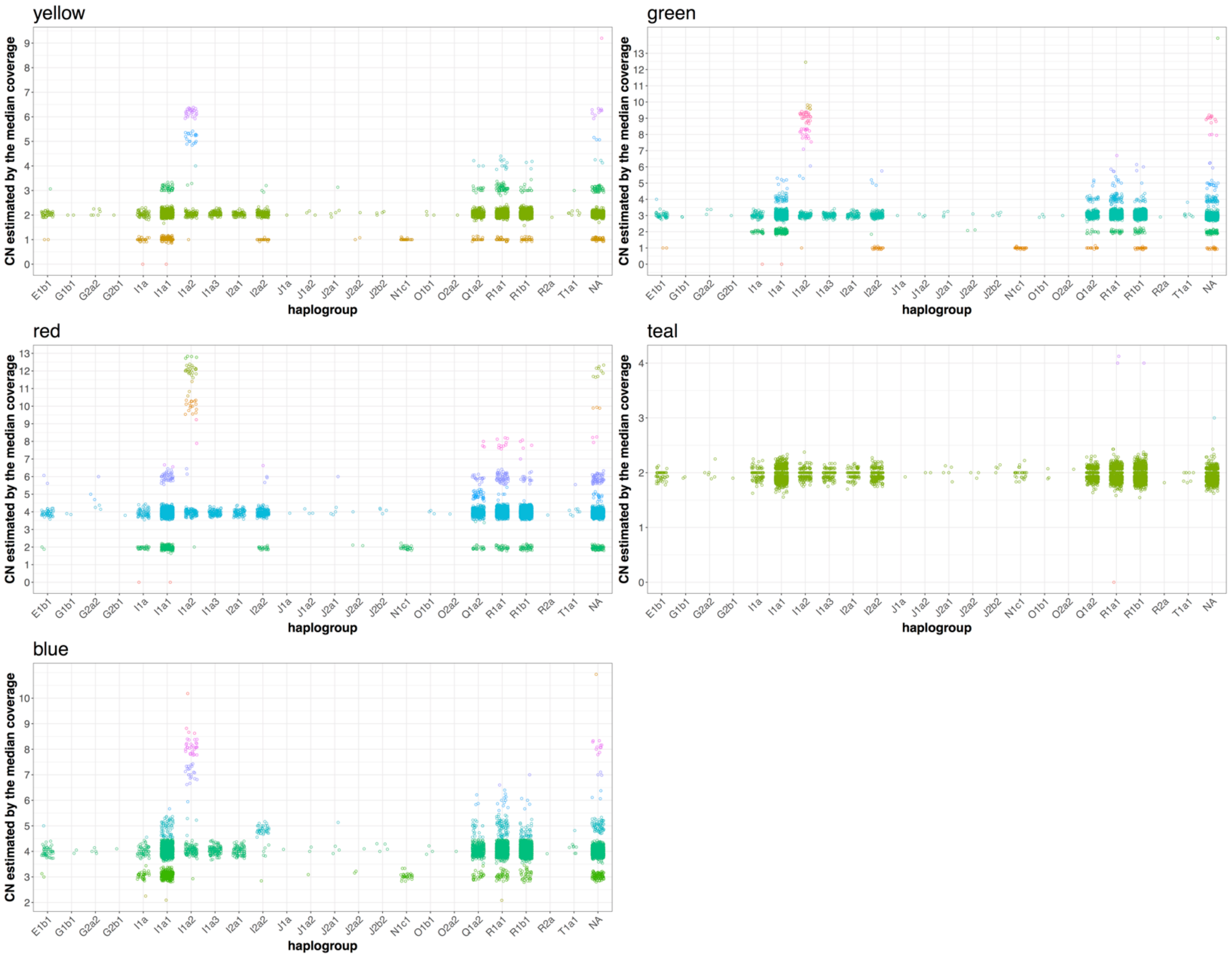

**Supplementary Figure 3: Copy number for palindromes 4, 5, 6 and 8** in each haplogroup of the Icelandic population estimated by the median of the median coverages over 1kb windows divided by the median coverage of single copy genes. Each dot represents an individual and is coloured according the copy number estimated using the HMM. Individuals with ambiguous copy number are not represented.

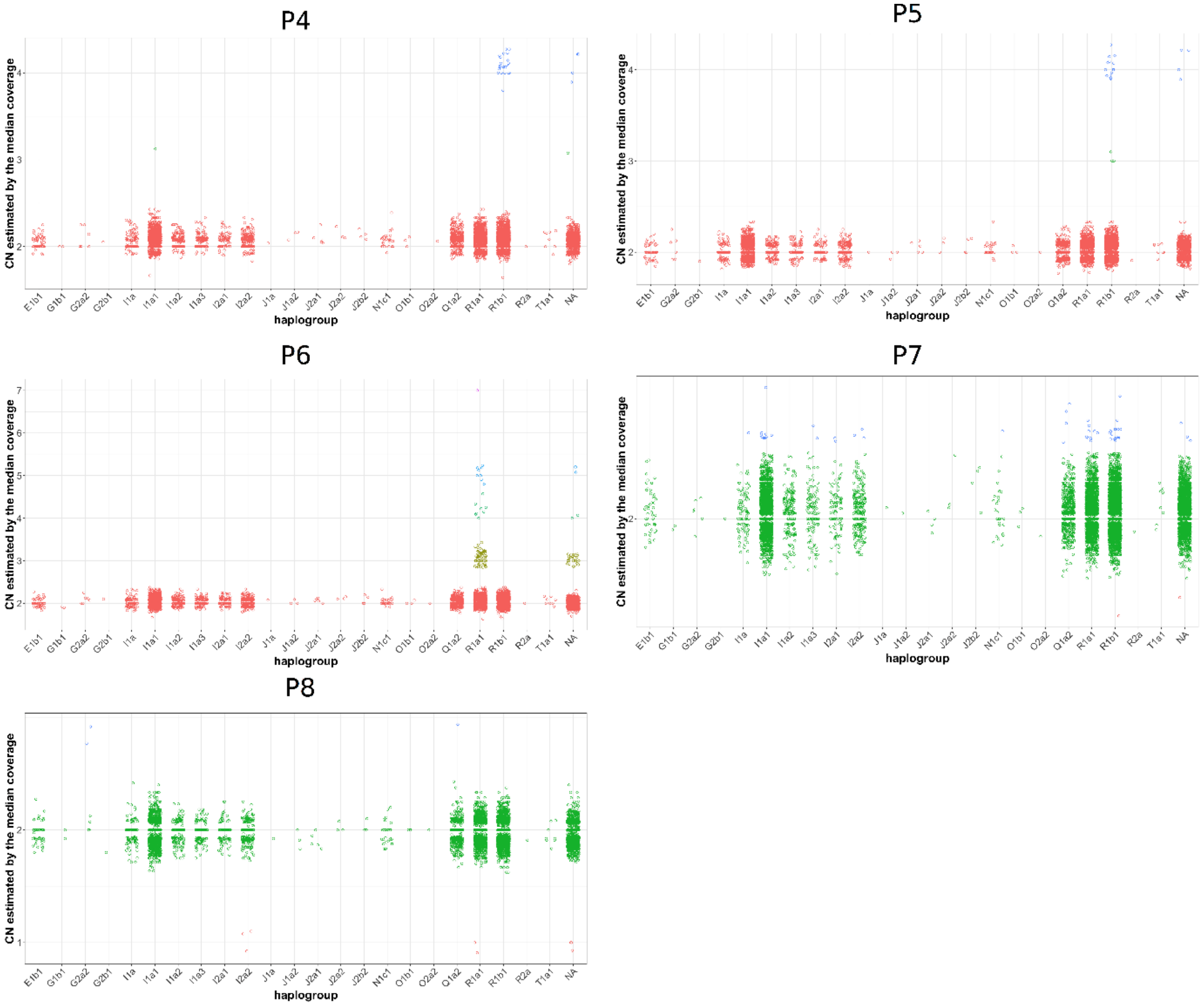

**Supplementary Figure 4: Example of individuals bearing the partial deletion of palindrome 1. A- For the 23 individuals from the same patriline,** copy number over non-overlapping 1kb windows for amplicon yellow, green and red. The position of each amplicon is indicated above the plots by coloured rectangles. **B- For the last individual represented in A**, copy number over non-overlapping 10kb windows as estimated on the unfolded Y reference.

A

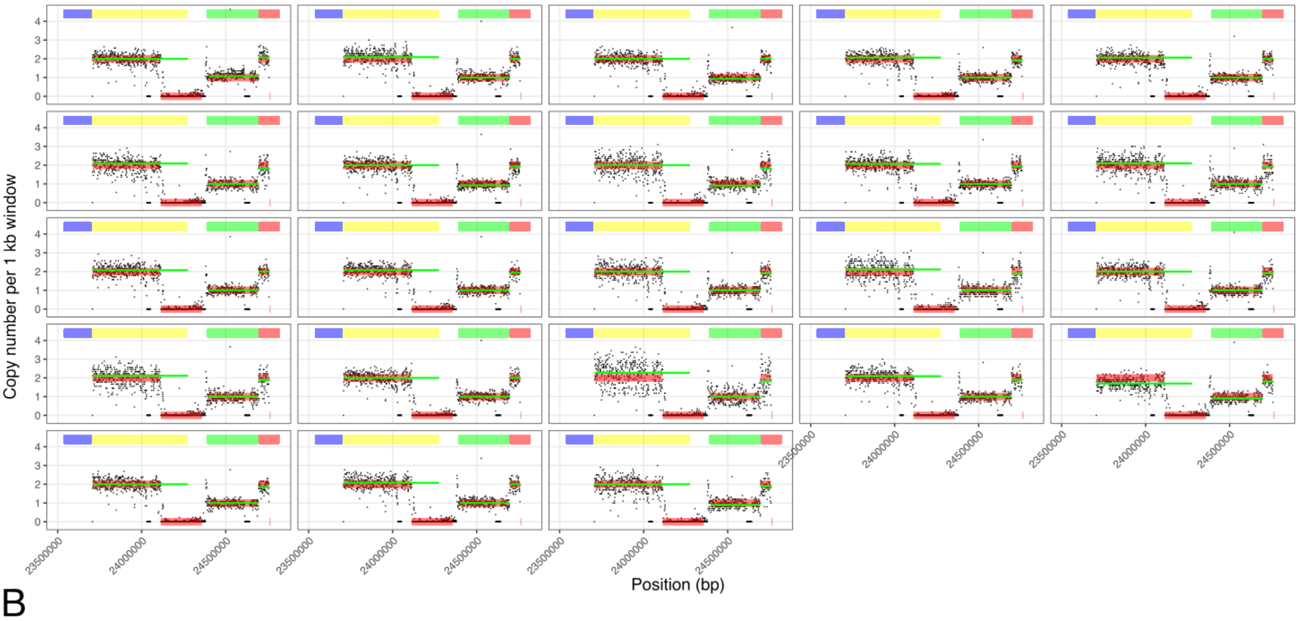


B

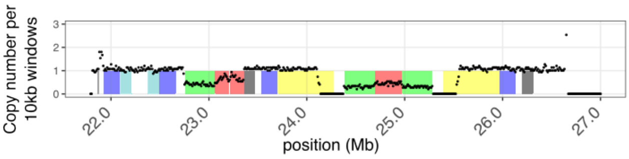

**Supplementary Figure 5: Previously reported deletions and amplifications in the AZFc region,** represented on the PCA of yellow, green and red amplicon copy number estimated by the median of the median coverage over 1kb windows over the amplicons. Each dot represents one individual, and is coloured according to the event: AZFc (red triangles), b1/b3 deletion (red cross), b2/b3 deletion (red x), b2/b3 or g1/g3 duplication (turquoise lozenge), b2/b4 duplication (green inverted triangles), gr/gr deletion (orange square), gr/gr duplication (green star), gr/gr deletion then b2/b4 duplication (dark blue lozenge), unknown events (grey star), reference CN (grey circle), and ambiguous copy number (pink circles).

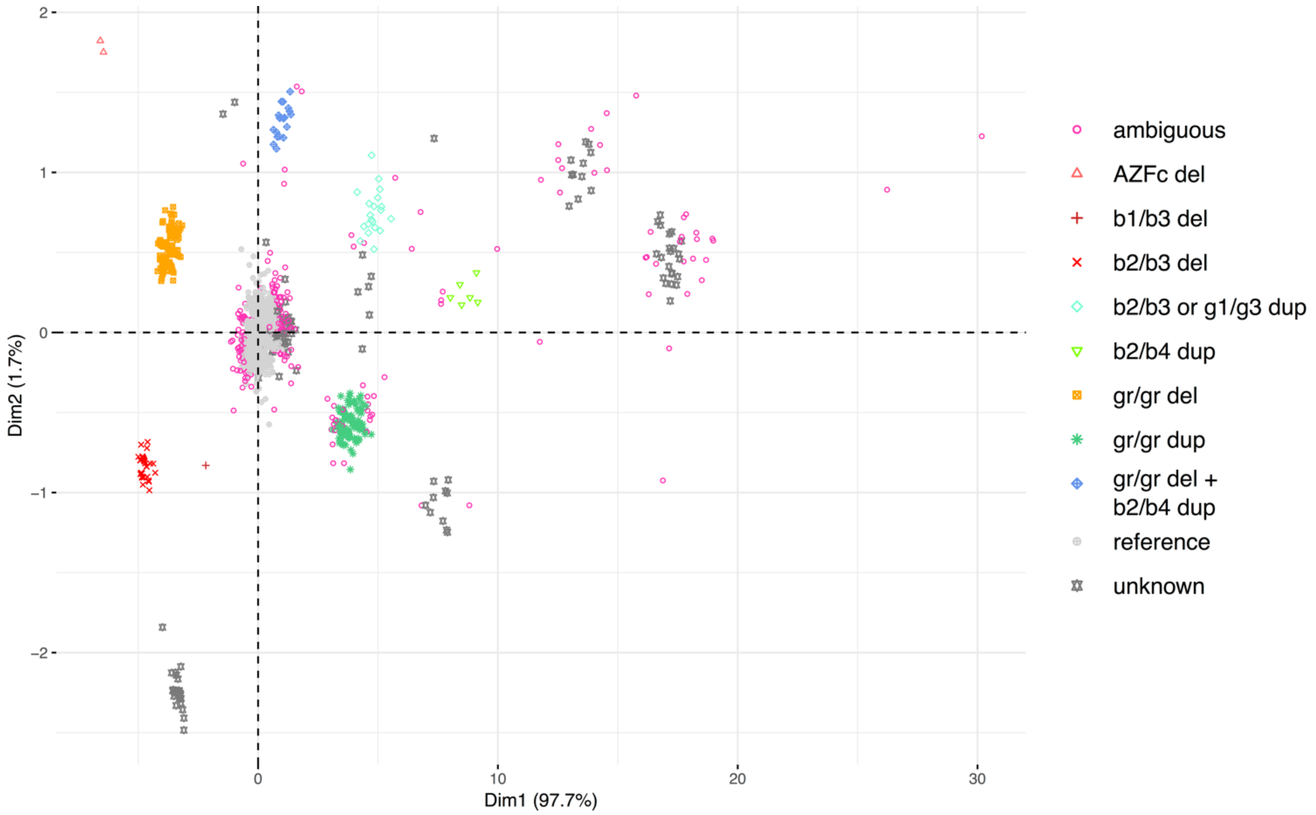

**Supplementary Figure 6: Copy number of A- green and yellow; B- red and; C- red and green,** coloured according to their clusters.

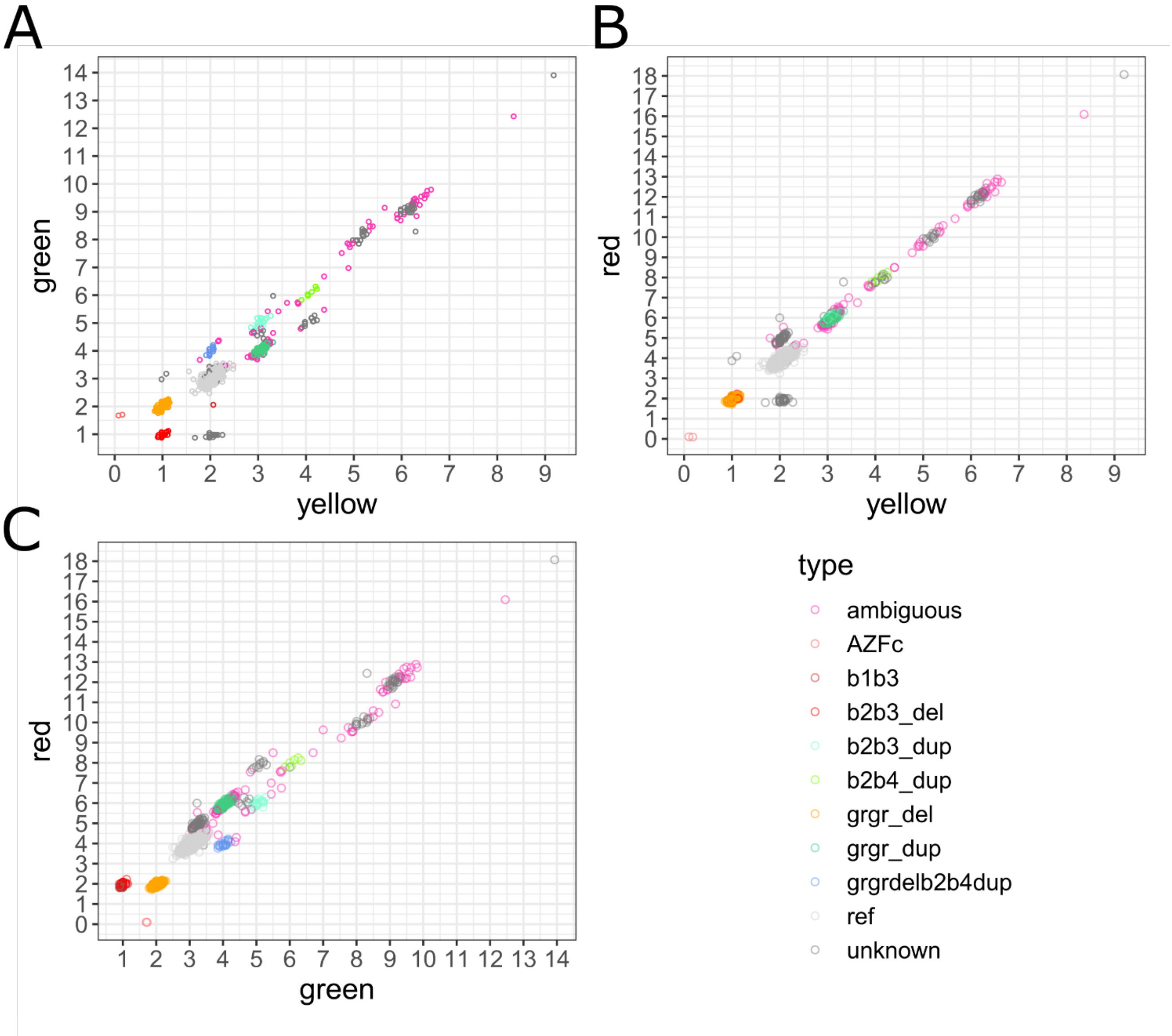

**Supplementary Figure 7: A- PCA constructed from the copy number of yellow, green and red.** Individuals are coloured according to their cluster. **0**: Unattributed, **1**: reference CN, **2**: gr/gr deletion, **3**: b2/b3 or g1/g3 duplication (higher cluster) and gr/gr duplication (lower cluster), **4**: b2/b3 deletion, **5**: unknown duplication, **6**: unknown deletion, **7**: unknown duplication, **8**: gr/gr deletion, **9**: unknown deletion, **10**: b2/b4 duplication, **11**: unknown duplication, **12**: b2/b4 deletion. **B- Description of the clusters detected in A.** Unknown deletions are colored in blue and unknown duplications in green.

A

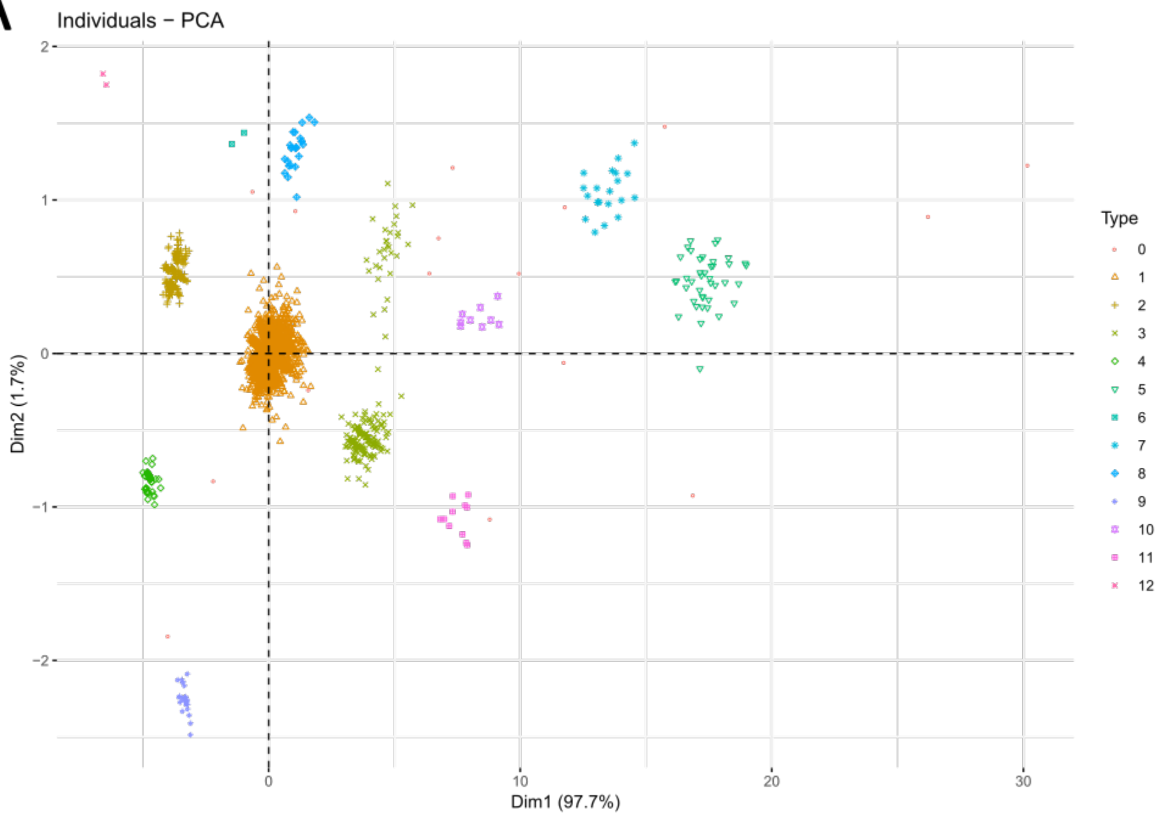


B

| cluster | Description | type | count | Copy number yellow | Copy number green | Copy number red | Copy number teal | Copy number blue |
|---|---|---|---|---|---|---|---|---|
| **1** | **reference** | **-** | **10579** | **2** | **3** | **4** | **2** | **4** |
| 2 | *gr/gr* del | deletion | 503 | 1 | 2 | 2 | 2 | 3 |
| 3 | *b2/b3* or *g1/g3* dup | duplication | 59 | 3 | 5 | 6 | 2 | 5 |
| | *gr/gr* dup | duplication | 149 | 3 | 4 | 6 | 2 | 5 |
| 4 | *b2/b3* del | deletion | 81 | 1 | 1 | 2 | 2 | 3 |
| 5 | unknown1 | duplication | 41 | 6 | 9 | 12 | | |
| 6 | unknown2 | deletion | 2 | 1 | 3 | 4 | | |
| 7 | unknown3 | duplication | 21 | 5 | 8 | 10 | | |
| 8 | *gr/gr* del + *b2/b4* dup | mixed | 24 | 2 | 4 | 4 | 2 | 4 |
| 9 | unknown4 | deletion | 28 | 0 | 1 | 2 | | |
| 10 | *b2/b4* dup | duplication | 9 | 4 | 6 | 8 | 2 | 6 |
| 11 | unknown5 | duplication | 11 | 4 | 5 | 6 | | |
| 12 | *b2/b4* del | deletion | 2 | 0 | 0 | 0 | 2 | 1 |

**Supplementary Figure 8**: **Boxplot of the distribution of copy number calculated over 1kb windows over each amplicon for each individual in patrilines where an event was detected.** Six examples are presented, three MD and three SD events. The number of measures for each individual corresponds to the length of the amplicon / 1000. Pink background indicates that an event was detected in this amplicon, while blue background indicates no event was detected. The type of the event is annotated on the top of each plot.

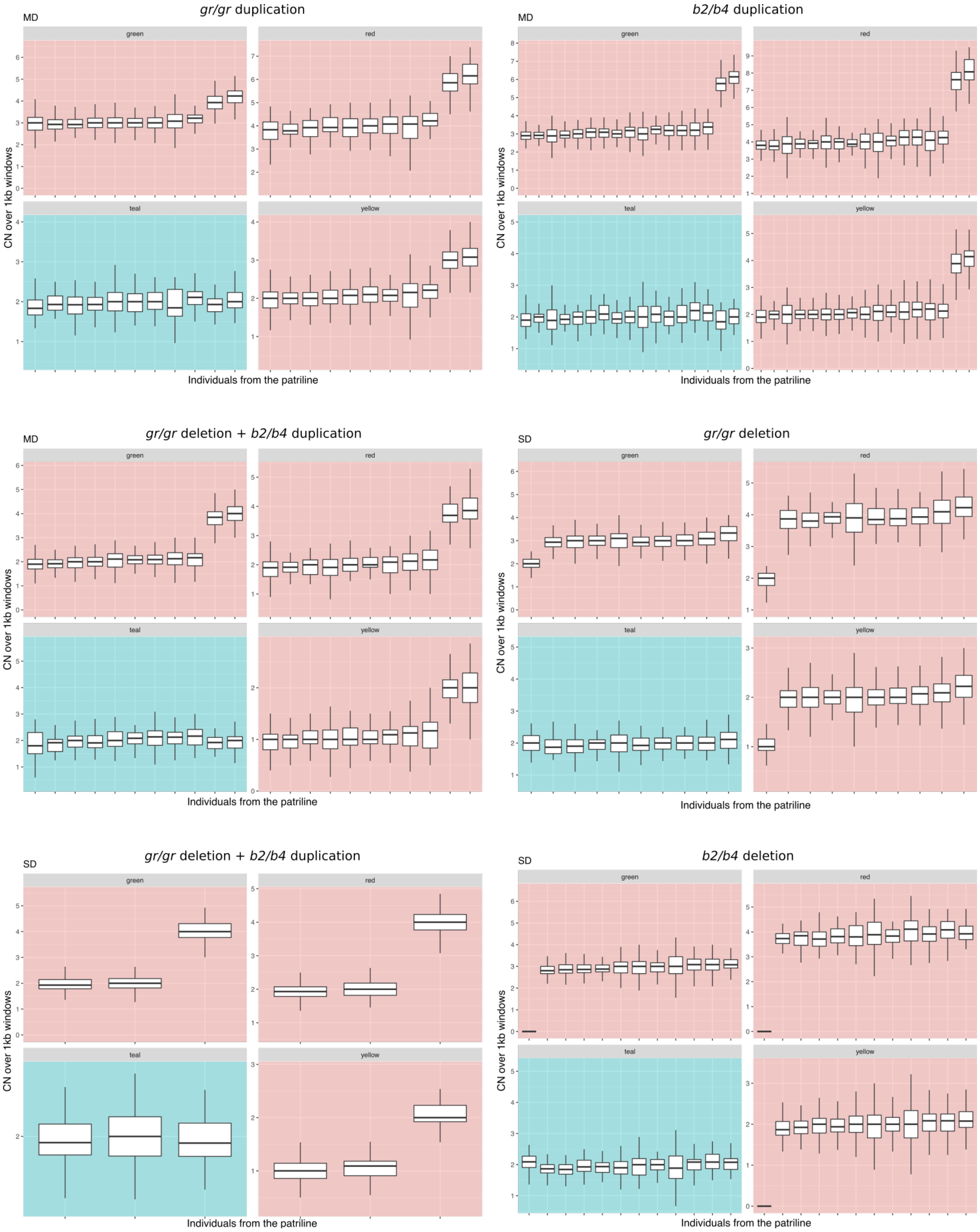
MD
gr/gr duplication
green
red
teal
yellow
CN over 1kb windows
Individuals from the patriline
MD
b2/b4 duplication
green
red
teal
yellow
CN over 1kb windows
Individuals from the patriline
MD
gr/gr deletion + b2/b4 duplication
green
red
teal
yellow
CN over 1kb windows
Individuals from the patriline
SD
gr/gr deletion
green
red
teal
yellow
CN over 1kb windows
Individuals from the patriline
SD
gr/gr deletion + b2/b4 duplication
green
red
teal
yellow
CN over 1kb windows
Individuals from the patriline
SD
b2/b4 deletion
green
red
teal
yellow
CN over 1kb windows
Individuals from the patriline

**Supplementary Figure 9: Example of an event of amplicon rescue, a duplication from 1 to 2 copies of palindrome 1. A-** Schematics of the palindrome structure in individuals with one copy of P1 and two copies of P1. **B- D-** illustrate an individual with one copy of P1, **C-E** illustrate an individual with 2 copies of P1. **B-C-** Copy number over non-overlapping 1kb windows for amplicon yellow, green and red. The position of each amplicon is indicated above the plots by coloured rectangles. **D-E-** Copy number over non-overlapping 10kb windows as estimated on the unfolded Y reference. **A-** The individuals with 1 copy are missing amplicon red in palindrome 1, and the region in the red box was duplicated in the individual with 2 copies. This scenario explains the copy number pattern of the amplicon seen in **B-C-D-E**.

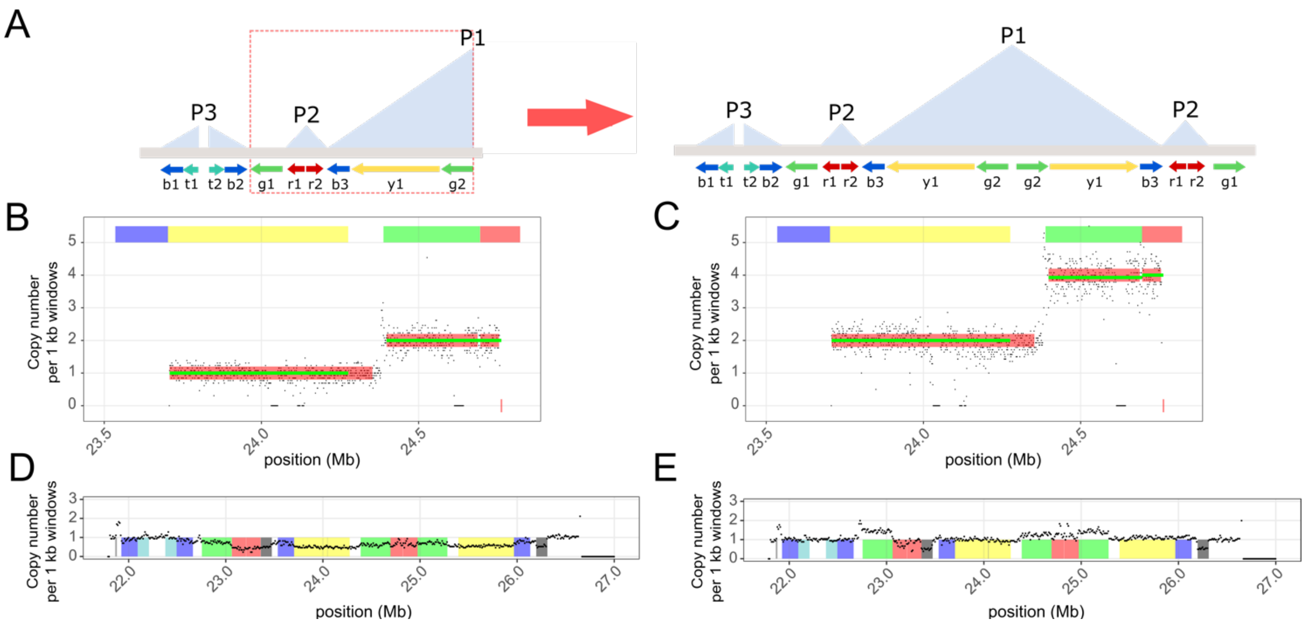

**Supplementary Figure 10- Mean number of children per birth year** for individuals carrying **A-** **r**eference (black) vs non-reference CN (yellow), **B-** reference (black) vs deletion (red) and duplication (blue), **C-** reference (black) vs non-reference CN (yellow) after Z-transformation, **D-** reference (black) vs deletion (red) and duplication (blue) after Z-transformation. The bars represent the 95% confidence interval for each calculated mean (dot). The number of samples is 11527 males.

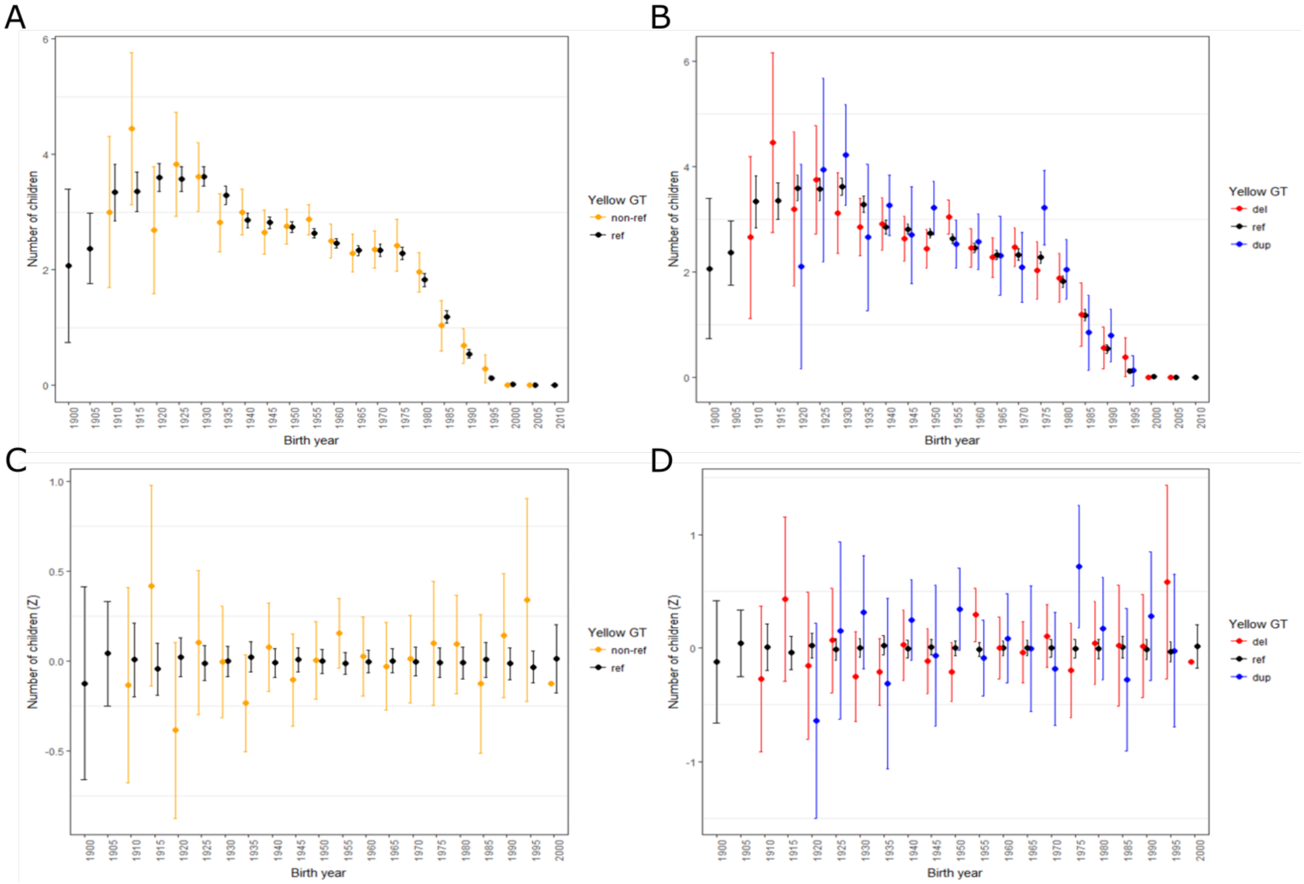

**Supplementary Figure 11: Simulation results, evolution of the proportions of individuals bearing non-reference CN over 500 generations** for a selection coefficient (s) between 0.00, and 0.03. The bold line represent the mean and the ribbons represent the standard deviation. The solid vertical lines represent the observed proportion in Iceland, and the dotted vertical lines represent the proportions at equilibrium under no selection.

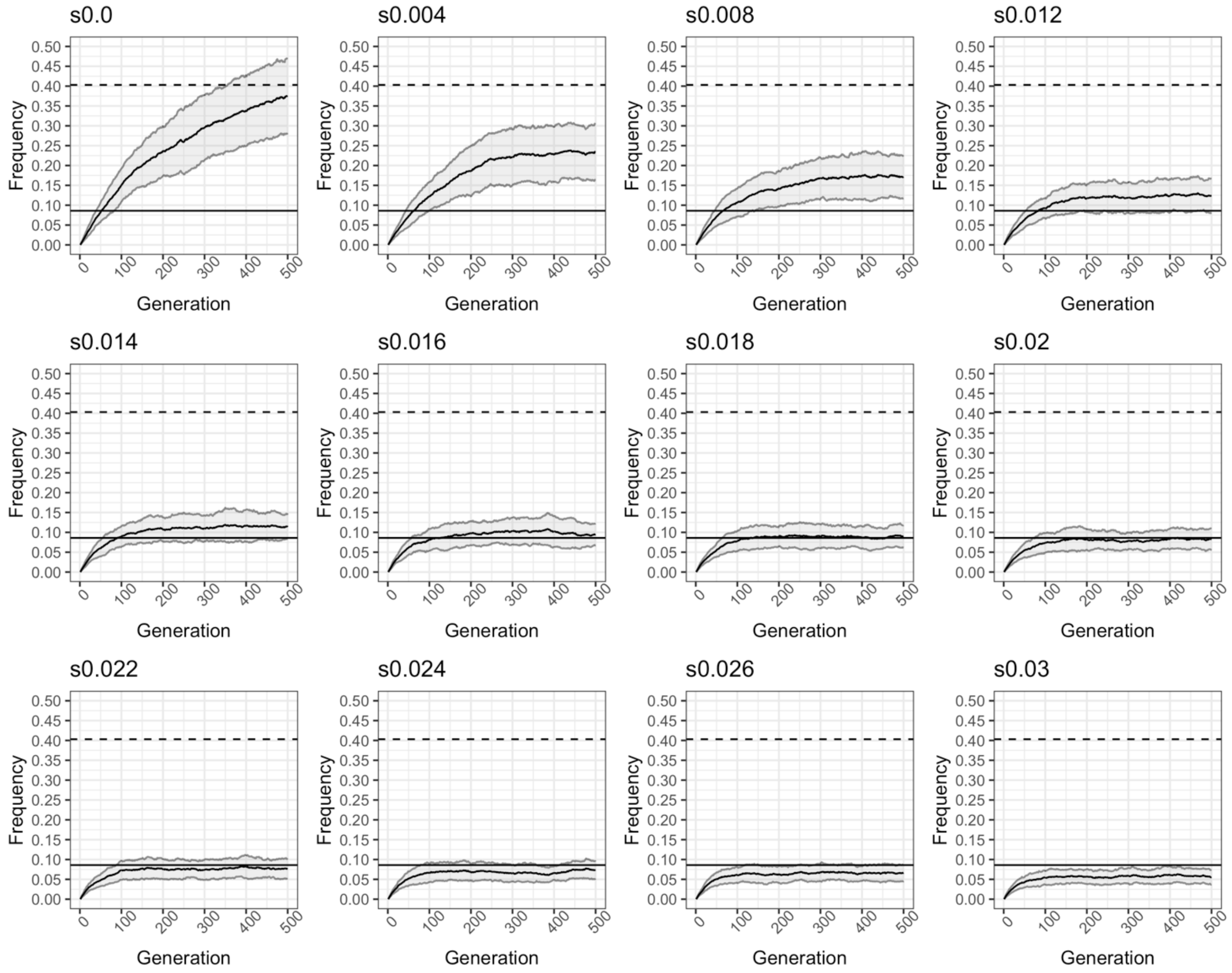

**Supplementary Figure 12: Distribution of the difference between the simulated mean proportions of individuals bearing non-reference CN over the 100 repetitions and the observed proportion in Iceland**. The number of values summarized by each boxplot is 400 generations (from 100th to 500th generation).

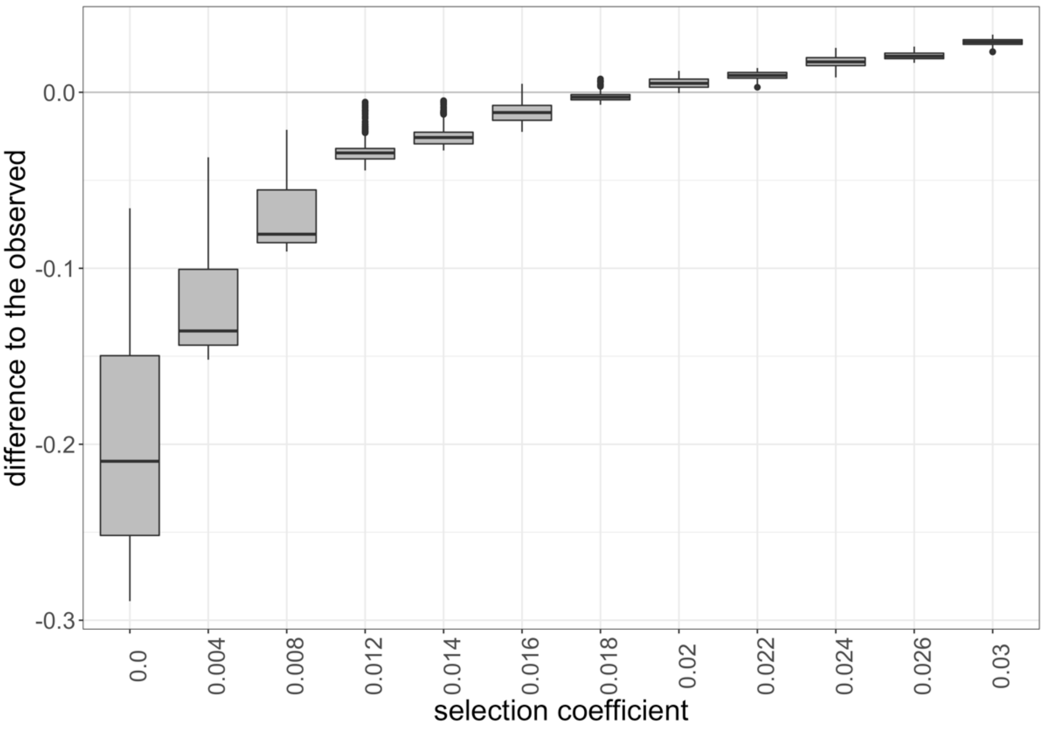

**Supplementary Figure 13**: **Power calculations A-** For a fixed selection coefficient (s=0.018) and a varying population size. The x axis is the population size, and the proportion of individuals carrying the reference CN and non-reference CN is the same than in the Icelandic population ($N_0$=91.4%*N vs $N_1$=8.6%*N). **B-** For a fixed $N_0$ (10460) and $N_1$ (927) corresponding to the number of individuals for which we have the number of children. **C-** For a fixed $N_0$ (7179) and $N_1$ (91), corresponding to the number of individuals included in the patrilines ($N_0$) and that show a de novo event ($N_1$). For each repetition, $N_0$ and $N_1$ number of children are drawn from a Poisson distribution of lambda 2.44 and lambda 2.44-(2.44*s), respectively, and a two-sided Student t test is performed between both drawn distributions. For 1000 repetitions, the power corresponds to the percentage of t tests (two sided) with a significant p-value (p<0.05). The red dots are the observed $N_1$ for A (12,000) and s for B and C (s=0.018).

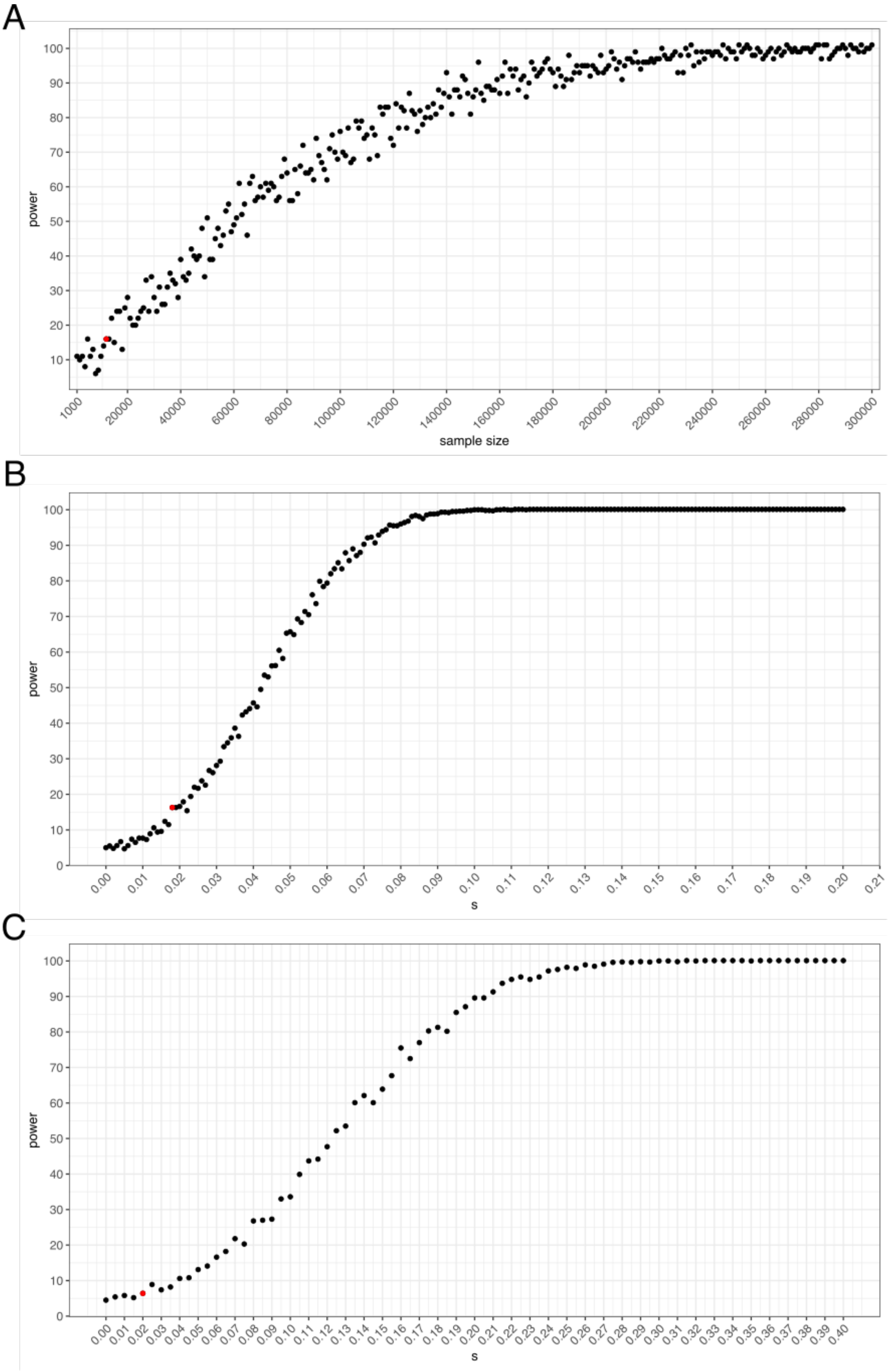
A
power
sample size
B
power
s
C
power
s

**Supplementary Figure 14:** Distribution of the differences between the CN estimates for the yellow amplicon using the set of single copy genes with TGIF2LY and PCDH11Y and without TGIF2LY and PCDH11Y.

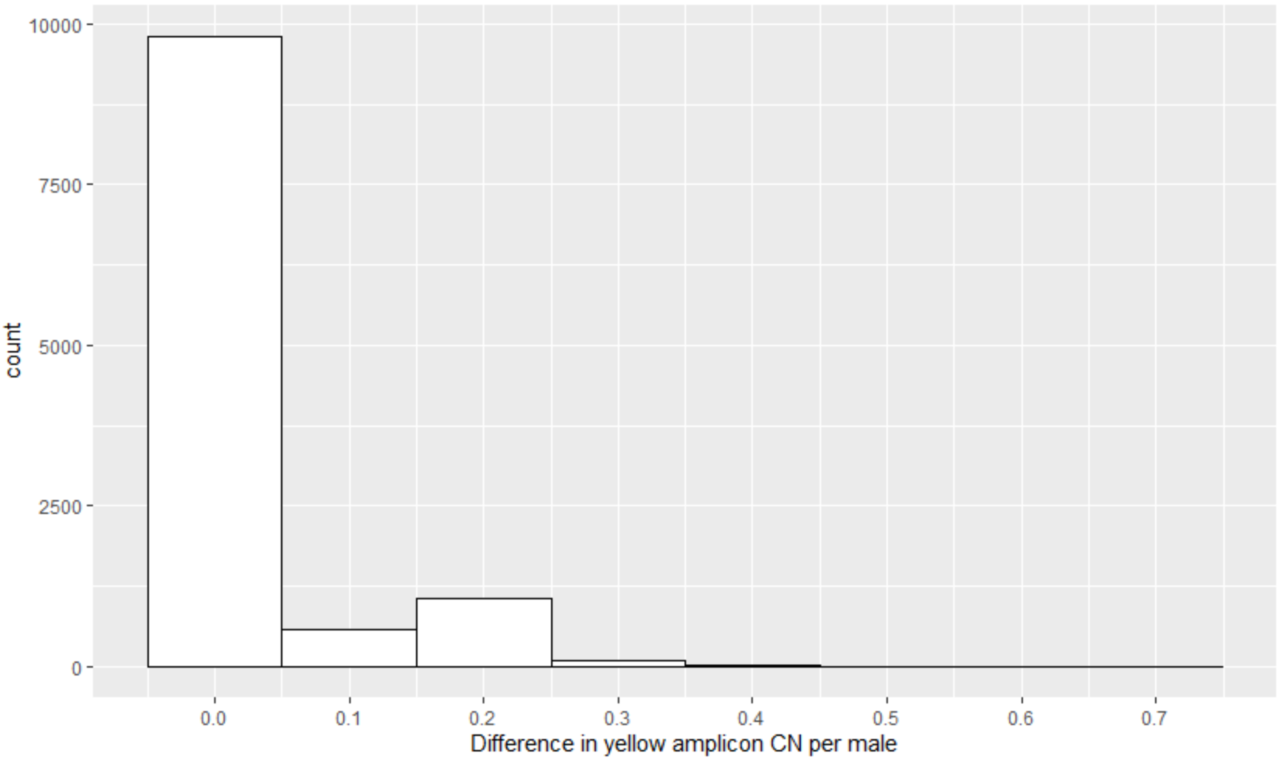

**Supplementary Figure 15: validation of breakpoints detected using the HMM**. Each panel is an individual. A and B are for the yellow amplicon, C and D are for the green amplicon. **A-C-** difference between the value of median CN calculated in the 1 kb window and the median CN of the cluster up (in blue), and the median CN of the cluster down (in red). **B-D-** ratio of the number of time the cluster up was closer to the observed value and the number of time the cluster down was closer to the observed value, for the segment identified with the HMM. **A and B**- the green boxes indicate the breakpoints that were validated (ratio is lower or equal to 0.25 or higher or equal to 0.75), **C and D-** the red boxes indicate the breakpoint that were not validated (ratio is higher than 0.25 and lower than 0.75).

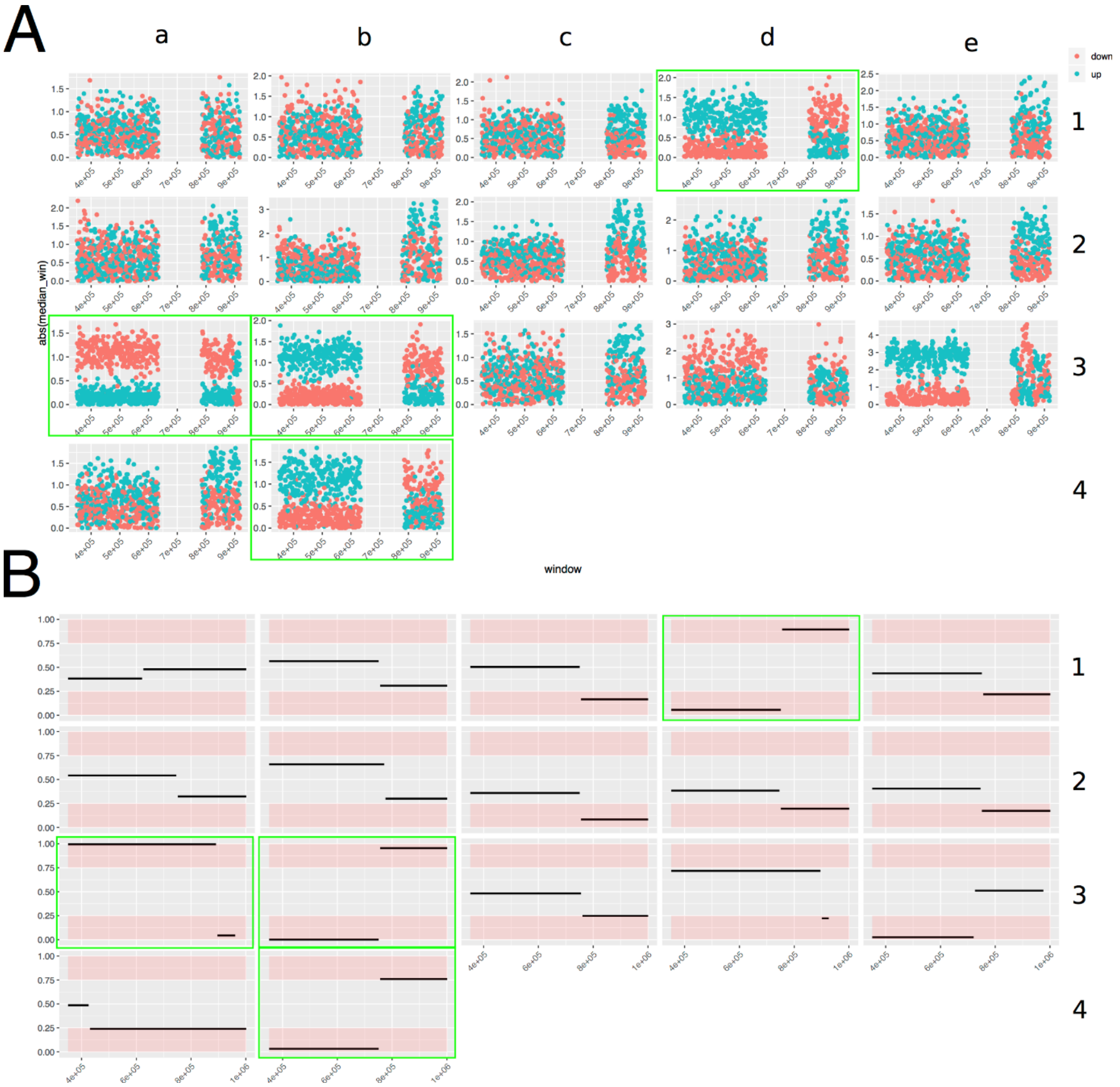

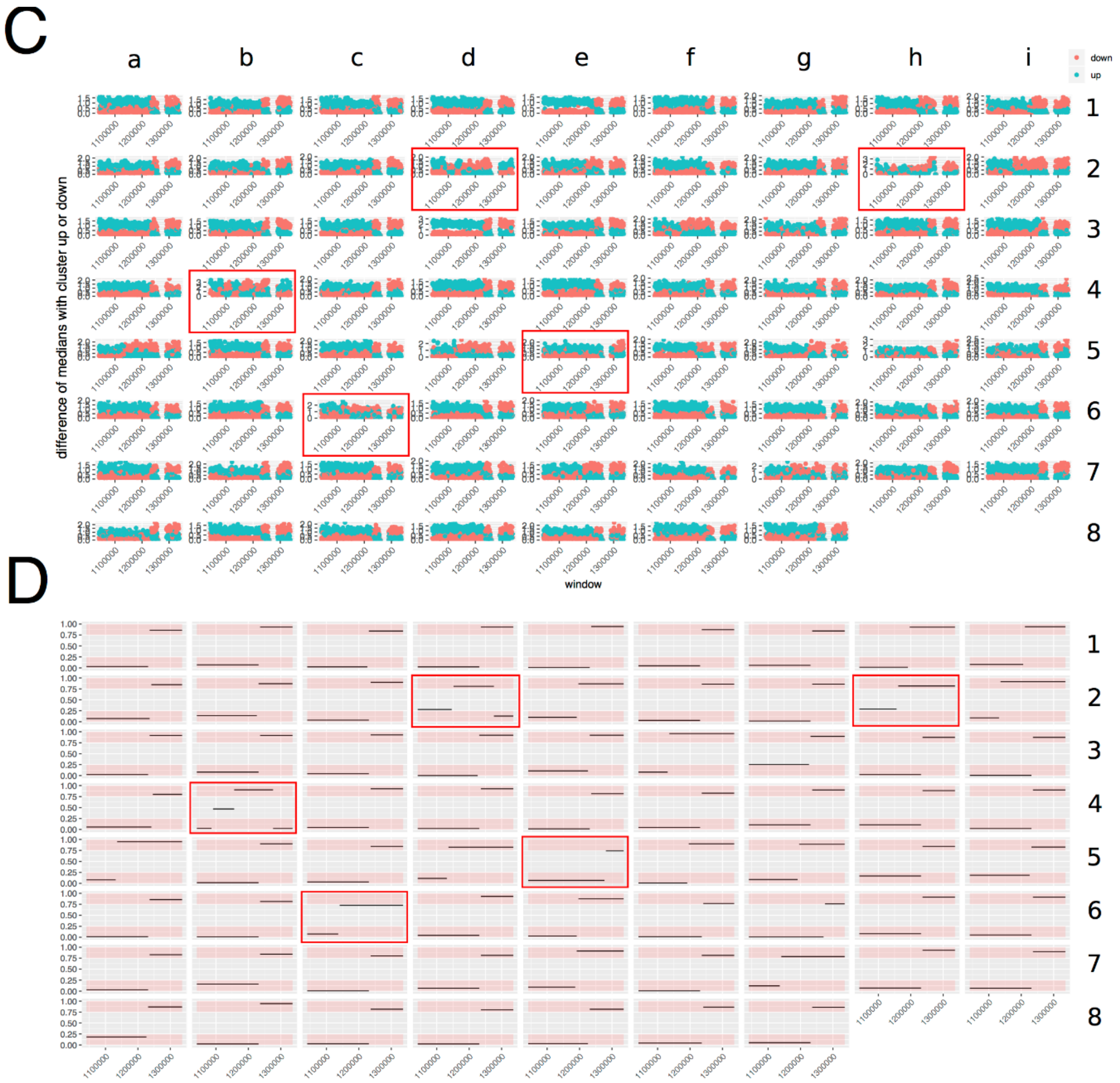
C
a
b
c
d
e
f
g
h
i
down
up
1
2
3
4
5
6
7
8
difference of medians with cluster up or down
window
1100000
1200000
1300000
D
1.00
0.75
0.50
0.25
0.00

**Supplementary Figure 16: standard deviation divided by the median copy number estimated in 1kb windows within each amplicon, in relation to the median copy number estimated over the whole amplicon.** Points are colored according to the copy number assigned to the individual. For individuals in red the difference between the CN estimated with the HMM and the CN estimated with the median coverage of the whole amplicon is superior or equal to 0.5.

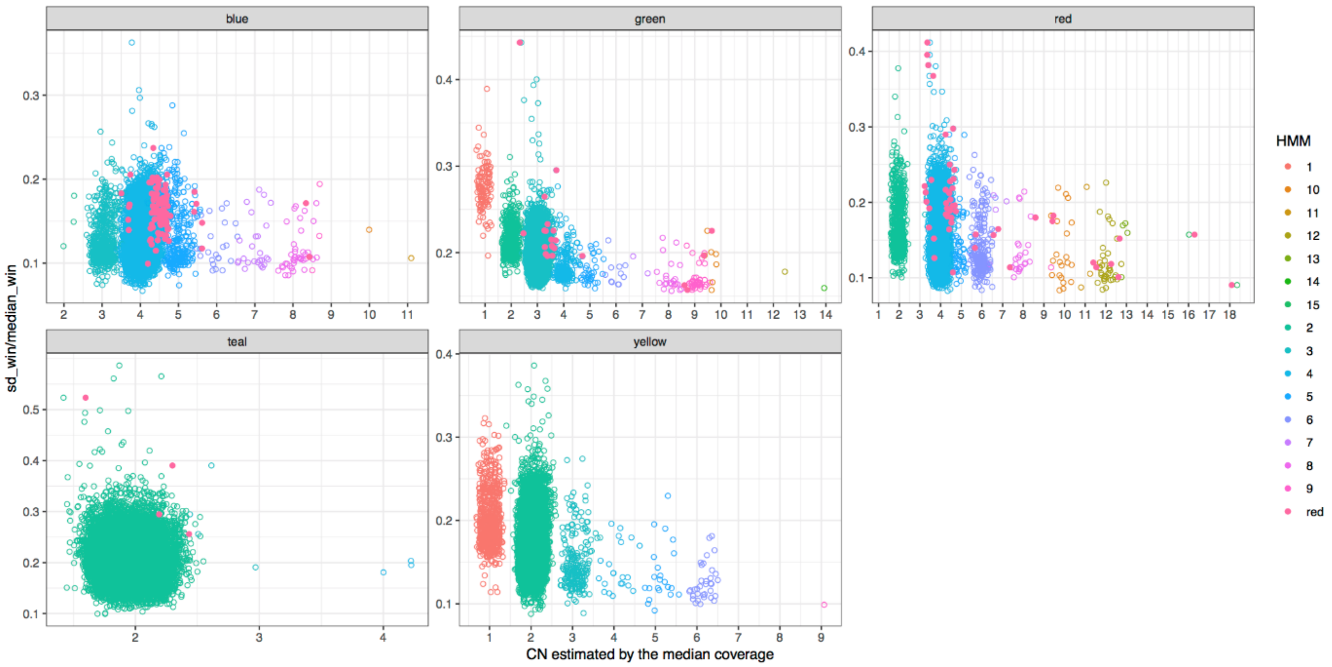

**Supplementary Figure 17: Individuals with ambiguous CN within the CN distribution estimated by the median of the medians of the coverage within non-overlapping 1 kb windows** for **A-** the amplicons within palindrome 1, 2 and 3, **B**- palindrome 4, 5, 6 and 8**.** Points are colored according to the copy number assigned to the individual. Individuals in red show a difference in CN estimates between the HMM and the median CN calculated over the whole amplicon superior or equal to 0.5. Individuals in grey are located outside of the boundaries of confidence of their cluster or were part of a CN cluster with less than 5 individuals and were assigned an ambiguous CN.

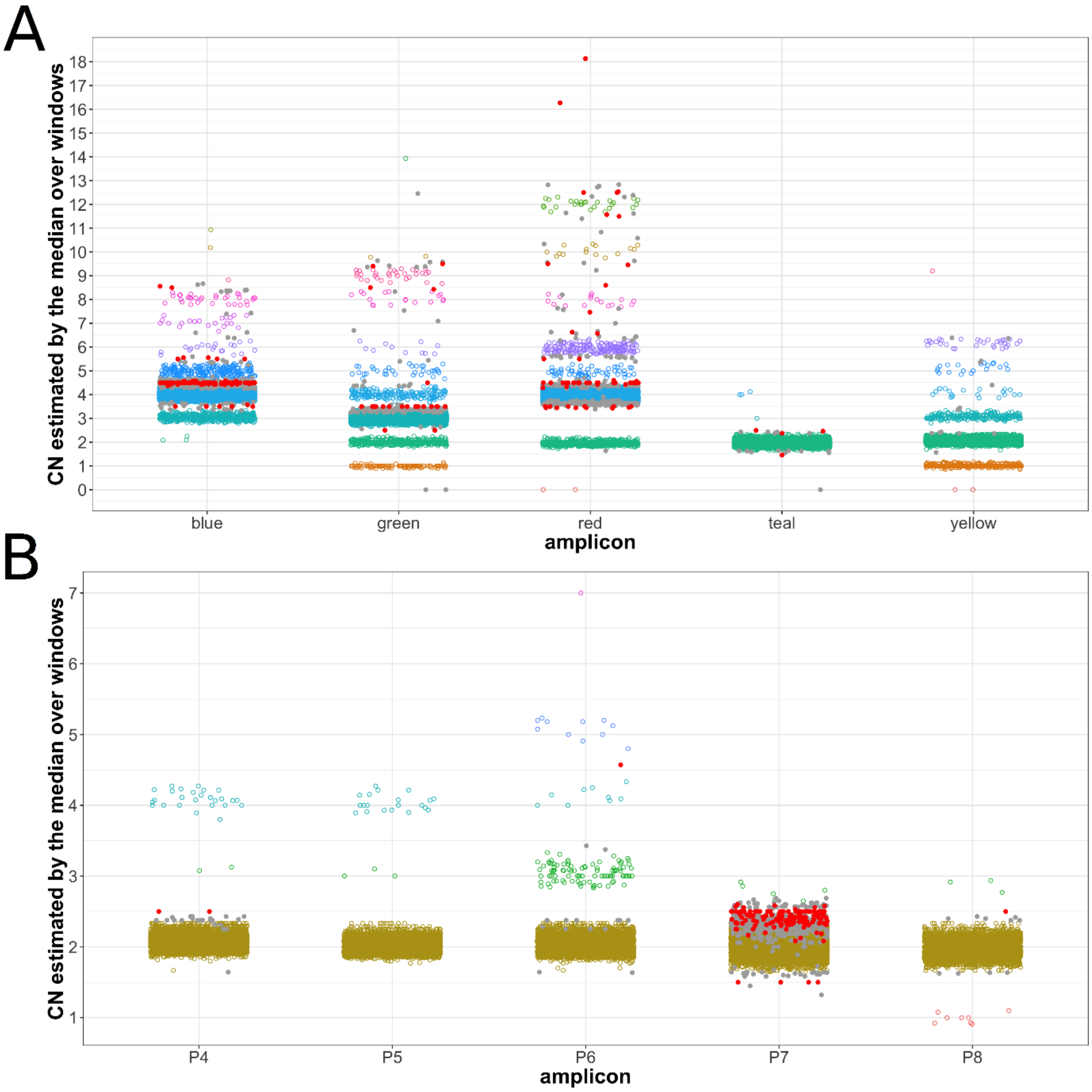

**Supplementary Figure 18: Differences in CN estimated by the median coverage over the whole amplicon between duplicates** for both types of duplicates: different samples (blood and buccal) and same sample (same tissue), for each amplicon and palindrome. N=77 for the category "different samples" and N=14 for "same samples".

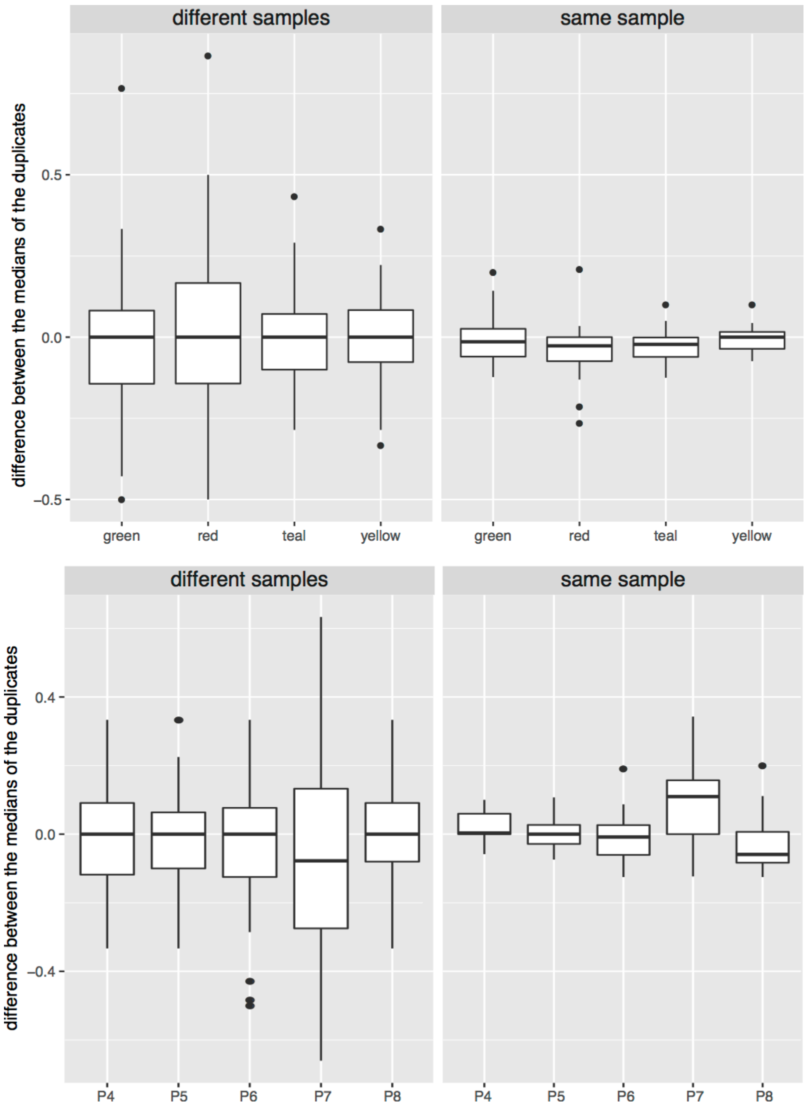

**Supplementary Figure 19**: **Distribution of the CN estimates for sliding non-overlapping windows of 1kb for individuals used as validation sample**. Each boxplot corresponds to one individual, in green and red are the individuals originally included in our analyses, the red being the individual bearing a singleton *de novo* event that is being tested for validation. Blue individuals were not originally included in the study, and were tested to validate the singleton *de novo* event detected in the red individuals. The blue individuals were not originally included because they were either sequenced after the freeze of the date (purple labels), or were sequenced with a technology non-PCR free (green label). The number of measures for each individual is the length of the amplicon / 1000.

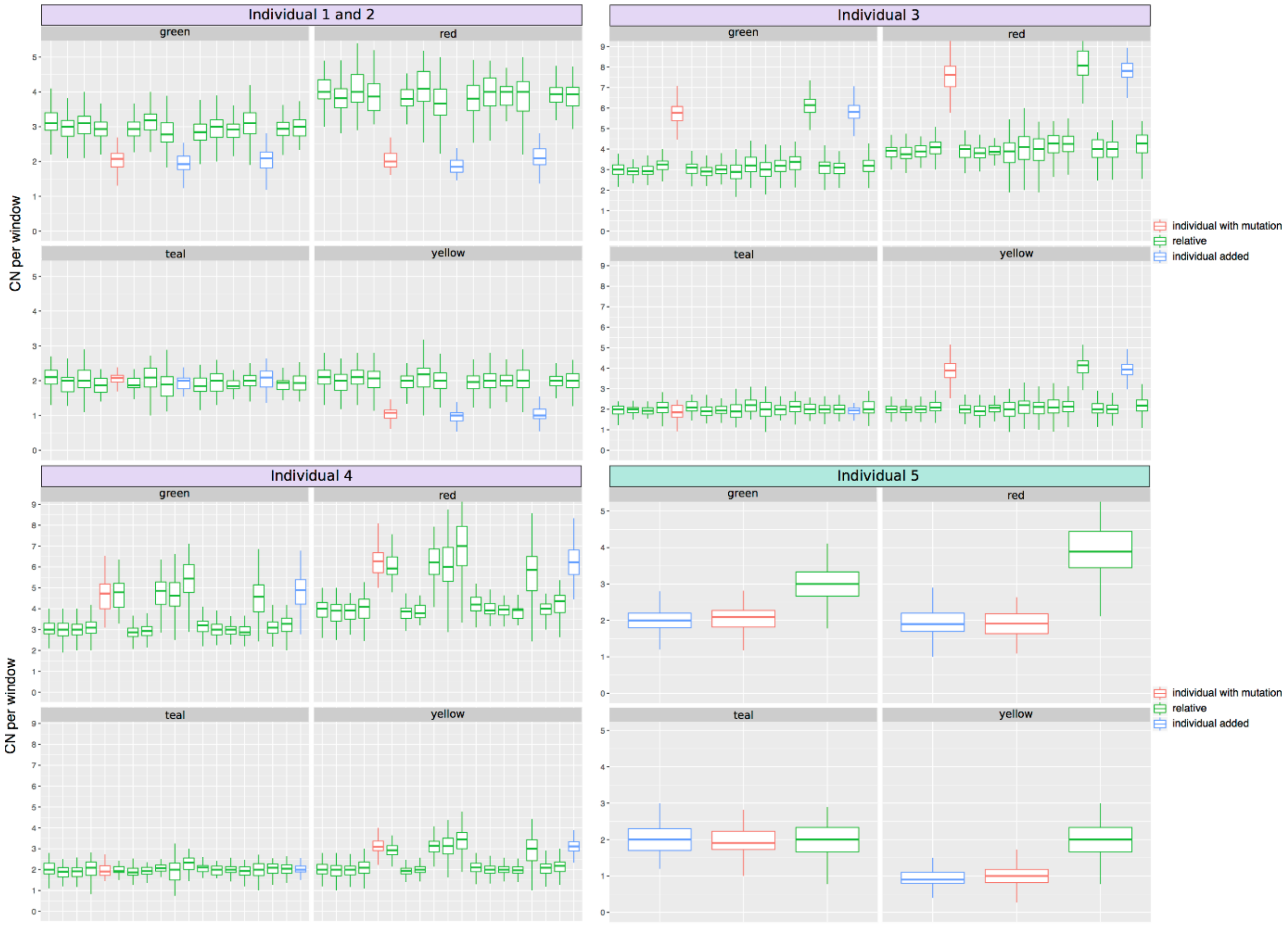

## SUPPLEMENTARY TABLES

**Supplementary Table 1: Genomic positions of the palindromic arms** included in the reference sequence constructed in hg38 coordinates.

| palindrome | type | hg38.start | hg38.end | length |
|---|---|---|---|---|
| P1 | proximal.arm | 23356858 | 24824727 | 1467870 |
| P3 | proximal.arm | 21904889 | 22208735 | 303847 |
| P4 | proximal.arm | 18430214 | 18754061 | 323848 |
| P5 | proximal.arm | 17452347 | 17954719 | 502373 |
| P6 | proximal.arm | 16139395 | 16289395 | 150001 |
| P7 | proximal.arm | 15862230 | 15885378 | 23149 |
| P8 | proximal.arm | 13981005 | 14023073 | 42069 |

**Supplementary Table 2: Genomic positions of the genes** included in the reference in hg38 coordinates.

| gene | Palindromes | Class | Gametolog | start | end | length |
|---|---|---|---|---|---|---|
| AMELY | - | X-degenerate | AMELX | 6863918 | 6913937 | 50019 |
| DDX3Y | - | X-degenerate | DBX | 12902108 | 12922478 | 20370 |
| EIF1AY | - | X-degenerate | EIF1AX | 20573711 | 20595154 | 21443 |
| KDM5D | - | X-degenerate | SMCX | 19703415 | 19746939 | 43524 |
| NLGN4Y | - | X-degenerate | NLGN4X | 14520608 | 14846945 | 326337 |
| PCDH11Y | - | X-transposed | PCDH11X | 4998226 | 5744228 | 746002 |
| PRKY | - | X-degenerate | PRKX | 7271972 | 7383547 | 111575 |
| RPS4Y1 | - | X-degenerate | RPS4X | 2839582 | 2868956 | 29374 |
| RPS4Y2 | - | X-degenerate | RPS4X | 20754068 | 20783056 | 28988 |
| SRY | - | X-degenerate | SOX3 | 2784855 | 2789741 | 4886 |
| TBL1Y | - | X-degenerate | TBL1X | 6908686 | 7093749 | 185063 |
| TGIF2LY | - | X-transposed | TFIG2LX | 3577085 | 3582041 | 4956 |
| TMSB4Y | - | X-degenerate | TMSB4X | 13701567 | 13708024 | 6457 |
| TXLNGY | - | X-degenerate | CXorf15 | 19565358 | 19609170 | 43812 |
| USP9Y | - | X-degenerate | USP9X | 12699231 | 12862844 | 163613 |
| UTY | - | X-degenerate | UTX | 13231920 | 13482670 | 250750 |
| ZFY | - | X-degenerate | ZFX | 2932416 | 2984508 | 52092 |
| BPY2 | P1 and P2 | Ampliconic | - | 22982263 | 23007465 | 25202 |
| CDY | P1 and P5 | Ampliconic | - | 25620115 | 25636745 | 16630 |
| DAZ | P1 and P2 | Ampliconic | - | 23127355 | 23201123 | 73768 |
| HSFY | P4 | Ampliconic | HSFX | 18544685 | 18590963 | 46278 |
| PRY exon1-2 | P1 | Ampliconic | - | 22482280 | 22492500 | 10220 |
| PRY all exons | P3 | Ampliconic | - | 22495000 | 22517543 | 22543 |
| RBMY1A1 | P3 and IR2 | Ampliconic | RBMX | 21532902 | 21550000 | 17098 |
| TSPY | TSPY array | Ampliconic | - | 9464955 | 9471749 | 6794 |
| XKRY | P5 | Ampliconic | - | 17766980 | 17772560 | 5580 |
| VCY | - | Ampliconic | VCX | 13983772 | 13988512 | 4740 |

**Supplementary Table 3: Genomic positions of the amplicons** within each palindromic arm, in hg38 coordinates.

| palindrome | amplicon | id | start | end | length |
|---|---|---|---|---|---|
| 3 | blue | b1 | 21925349 | 22093051 | 167703 |
| 3 | teal | t1 | 22093051 | 22208739 | 115689 |
| 1 | green | g2 | 24388041 | 24695116 | 307076 |
| 1 | red | r3 | 24695499 | 24822589 | 127091 |
| 1 | yellow | y1 | 23702730 | 24276001 | 573272 |

**Supplementary Table 4: Number of individuals with an ambiguous copy number** assigned using the breakpoint analysis, the cut-off, or belonging to an isolated cluster (<5 individuals), per region. The percentages are over the whole population (11,527 individuals).

| | Breakpoints | Cut-off | Isolated cluster | Total | Percentage |
|---|---|---|---|---|---|
| yellow | 22 | 13 | 0 | 35 | 0.30 |
| green | 5 | 162 | 2 | 169 | 1.47 |
| red | 2 | 354 | 8 | 364 | 3.16 |
| teal | 17 | 19 | 0 | 36 | 0.31 |
| blue | 262 | 393 | 0 | 755 | 6.55 |
| P4 | 16 | 26 | 0 | 42 | 0.36 |
| P5 | 80 | 0 | 0 | 80 | 0.69 |
| P6 | 11 | 16 | 0 | 27 | 0.23 |
| P7 | 3 | 647 | 0 | 650 | 5.64 |
| P8 | 6 | 11 | 0 | 17 | 0.15 |
| Total | 424 | 1641 | 0 | 2175 | |

**Supplementary Table 5: Summary of the copy number variations per region** (N=11,527). Each number is expressed as a copy number.

| AR | median | variance | minimum | maximum |
|---|---|---|---|---|
| yellow | 2.00 | 0.18 | 0.10 | 9.20 |
| green | 3.00 | 0.35 | 0.90 | 13.93 |
| red | 3.93 | 0.69 | 0.09 | 18.07 |
| teal | 2.00 | 0.01 | 1.14 | 4.13 |
| P4 | 2.07 | 0.02 | 1.57 | 4.27 |
| P5 | 2.00 | 0.01 | 1.71 | 4.27 |
| P6 | 2.00 | 0.03 | 1.64 | 6.91 |
| P8 | 2.00 | 0.01 | 0.91 | 2.94 |

**Supplementary Table 6: Number of individuals bearing the reference copy number, or bearing a variation in copy number (CNV)** in one or several palindromes, for palindromes 1, 2, 3. The breakpoint categories indicate events were the amplicon was partially duplicated or deleted.

| CNV | reference | P1+P2+P3 | P1+P2 | P2+P3 | P1 | P2 | P3 | green only | breakpoint green | breakpoint yellow | total |
|---|---|---|---|---|---|---|---|---|---|---|---|
| count | 10535 | 3 | 887 | 1 | 29 | 61 | 1 | 2 | 4 | 4 | 11527 |
| % | 91.39% | 0.03% | 7.69% | 0.01% | 0.25% | 0.53% | 0.01% | 0.02% | 0.03% | 0.03% | 100% |

**Supplementary Table 7: Number of individuals bearing the reference copy number, or bearing a variation in copy number (CNV)** in one or several palindromes, for palindromes 4, 5, 6, 8.

| CNV | reference | P4+P5 | P4 | P5 | P6 | P8 | total |
|---|---|---|---|---|---|---|---|
| count | 11374 | 21 | 1 | 1 | 117 | 11 | 11525 |
| % | 98.69% | 0.18% | 0.01% | 0.01% | 1.02% | 0.10% | 100% |

**Supplementary Table 8: Number of individuals bearing a CNV detected in palindrome 1, 2 or 3 and palindrome 4, 5, 6 or 8**. For example, 20 individuals show a CNV in P1 and P4 and P5.

| CNV | P4 and P5 | P6 | P8 |
|---|---|---|---|
| P1 | 20 | 0 | 0 |
| P2 | 0 | 16 | 2 |
| P3 | 0 | 1 | 0 |

**Supplementary Table 9- Mismatch count between the original CN and the validation analysis using an independent coverage estimation.** Summary statistics for relative sequence depth (rdepth) estimates per CN category. The 0 CN category contains 27 individuals that share the same (unknown) partial deletion of the yellow amplicon (see Figure S4), emphasized by their median values reflecting the 2 CN category, whilst their mean values are in line with the 1 CN category. For this reason we report the median and mean values excluding the 0 CN category. The remaining two individuals in the 0 CN category carry a b2/b4 deletion.

| original CN | N | rdepth Median rounded | | | | rdepth Mean rounded | | | |
|---|---|---|---|---|---|---|---|---|---|
| | | mismatch count | mismatch rate | avg Median | sd Median | mismatch count | mismatch rate | avg Mean | sd Mean |
| 0 | 29 | 27 | 0.931 | 1.862 | 0.516 | 27 | 0.931 | 0.966 | 0.325 |
| 1 | 482 | 0 | 0 | 1 | 0 | 0 | 0 | 1 | 0 |
| 1-2 | 2 | 0 | 0 | 2 | 0 | 0 | 0 | 2 | 0 |
| 1-4 | 1 | 0 | 0 | 1 | NA | 0 | 0 | 2 | NA |
| 2 | 8407 | 1 | 0 | 2 | 0.011 | 4 | 0 | 2 | 0.022 |
| 2-3 | 14 | 0 | 0 | 2 | 0 | 0 | 0 | 2 | 0 |
| 3 | 148 | 1 | 0.007 | 3.007 | 0.082 | 1 | 0.007 | 3.007 | 0.082 |
| 3-4 | 4 | 0 | 0 | 3 | 0 | 0 | 0 | 3 | 0 |
| 4 | 17 | 0 | 0 | 4 | 0 | 0 | 0 | 4 | 0 |
| 4-5 | 2 | 0 | 0 | 4 | 0 | 0 | 0 | 4 | 0 |
| 5 | 18 | 0 | 0 | 5 | 0 | 1 | 0.056 | 5.056 | 0.236 |
| 5-6 | 4 | 0 | 0 | 5 | 0 | 0 | 0 | 5 | 0 |
| 6 | 25 | 0 | 0 | 6 | 0 | 0 | 0 | 6 | 0 |
| 6-7 | 8 | 0 | 0 | 6 | 0 | 0 | 0 | 6.125 | 0.354 |
| 9-10 | 1 | 1 | 1 | 8 | NA | 1 | 1 | 8 | NA |
| Totals | 9162 | 30 | 0.003 | | | 34 | 0.004 | | |
| Totals excluding 0 copies | 9133 | 3 | 0.0003 | | | 7 | 0.001 | | |

**Supplementary Table 10– Comparison of Teitz et al. CN estimates and our CN estimates for 13 individuals from the 1000 Genomes project**, representing different haplogroups and CN estimates.

| ID | Haplogroup | Teitz et al. CN estimate | Our CN estimate |
|---|---|---|---|
| HG00159 | I1 | 2 | 1.964 |
| HG00182 | N1 | 1 | 1.053 |
| HG00183 | N1 | 1 | 1.001 |
| HG00242 | R1b | 2 | 1.993 |
| HG00244 | R1b | 2 | 2.013 |
| HG00290 | N1 | 1 | 0.987 |
| HG00338 | N1 | 1 | 1.003 |
| HG00345 | I1 | 2 | 2.043 |
| HG00371 | N1 | 1 | 1.014 |
| HG01867 | O2 | 5 | 5.085 |
| HG03445 | E1b | 6 | 6.036 |
| NA12546 | G2 | 4 | 3.918 |
| NA20758 | T | 3 | 2.953 |

**Supplementary Table 11– Mutation matrix observed** within the patrilines. The numbers in the diagonal are the number of times we have observed Y chromosome transmission without a change in CN.

| from/to | 0 | 1 | 2 | 2.0-3.0 | 2.5 | 3 | 3.0-4.0 | 3.5 | 4 | 4.0-5.0 | 5 | 6 | 6.0-7.0 |
|---|---|---|---|---|---|---|---|---|---|---|---|---|---|
| 0 | 62 | | | | | | | | | | | | |
| 1 | | 1194 | 3 | | | | | | | | | | |
| 2 | 2 | 16 | 22518 | | 1 | 20 | | 1 | 2 | | | | |
| 2.0-3.0 | | | | 18 | | | | | | | | | |
| 2.5 | | | | | 1 | | | | | | | | |
| 3 | | | 2 | | | 321 | | | | 1 | | | |
| 3.0-4.0 | | | | | | | 16 | | | | | | |
| 3.5 | | | | | | | | 0 | | | | | |
| 4 | | | | | | | | | 24 | | | | |
| 4.0-5.0 | | | | | | | | | | 0 | | | |
| 5 | | 1 | | | | | | | | | 59 | 1 | |
| 6 | | | | | | | | | | | 4 | 71 | |
| 6.0-7.0 | | | | | | 2 | | | 1 | | | | 53 |

**Supplementary Table 12– Mutation matrix observed** within the phylogenetic tree constructed with 891 individuals with at least 10 SNP differences.

| from/to | 0 | 1 | 2 | 3 | 4 | 5 | 6 |
|---|---|---|---|---|---|---|---|
| 0 | | | | | | | |
| 1 | | | 1 | 1 | | | |
| 2 | 2 | 22 | | 18 | 2 | | 1 |
| 3 | | | | | 1 | | |
| 4 | | | | | | | |
| 5 | | | | | | | |
| 6 | | | | | | | |

**Supplementary Table 13: Grouping of the events detected in patrilines in several amplicons.** GRY: green, red and yellow; RY: red and yellow; GRT: green, red and teal; GRYT: green, red, yellow and teal. The events are divided into multiple descendants (MD) and single descendants (SD) events; and into amplification (+), deletion (-) and not-determined (NA) events.

| type mut | type event | GRY | RY | GRT | Green | Red | GRYT | Yellow | total |
|---|---|---|---|---|---|---|---|---|---|
| MD | + | 12 | 0 | 0 | 0 | 0 | 0 | 1 | 13 |
| | - | 4 | 0 | 0 | 0 | 0 | 0 | 0 | 4 |
| | NA | 0 | 0 | 0 | 0 | 0 | 0 | 0 | 0 |
| | total | 16 | 0 | 0 | 0 | 0 | 0 | 1 | 17 |
| SD | + | 13 | 2 | 0 | 1 | 1 | 1 | 0 | 18 |
| | - | 24 | 0 | 1 | 2 | 0 | 0 | 0 | 27 |
| | NA | 0 | 0 | 0 | 0 | 0 | 0 | 0 | 0 |
| | Total | 37 | 2 | 1 | 3 | 1 | 1 | 0 | 45 |
| TOTAL | | 53 | 2 | 1 | 3 | 1 | 1 | 1 | 62 |

**Supplementary Table 14: Count of the PSVs in the yellow amplicon** and palindrome 1 (P1) for individuals belonging to the patrilines where a rescue event (from 1 to 2 copies) was detected and for four outgroup individuals with the same haplogroup. For each individual, the state of the copy number is represented (two copies with no event, single copy or two copies after duplication). The columns represent, for each genomic region, the count of valid genotypes at positions containing variation in our dataset (ValidGTCnt) and the count of PSVs with a pseudo-heterozygous genotype (hetGTCnt).

| Patriline | Individual | Haplogroup | Phylogenetic marker | Yellow hetGTCnt | P1 validGTCnt | P1 hetGTCnt |
|---|---|---|---|---|---|---|
| patriline 1 | single-copy | I1a1b3b | L813/S436/Z719 | 5 | 4202 | 2 |
| | 2 copies, duplication | I1a1b3b | L813/S436/Z720 | 4 | 4202 | 7 |
| | single-copy | I1a1b3b | L813/S436/Z721 | 4 | 4202 | 5 |
| | single-copy | I1a1b3b | L813/S436/Z722 | 4 | 4201 | 5 |
| | single-copy | I1a1b3b | L813/S436/Z723 | 4 | 4197 | 5 |
| | single-copy | I1a1b3b | L813/S436/Z724 | 4 | 4202 | 5 |
| | single-copy | I1a1b3b | L813/S436/Z725 | 4 | 4202 | 5 |
| | single-copy | I1a1b3b | L813/S436/Z726 | 6 | 4195 | 6 |
| | 2 copies, duplication | I1a1b3b | L813/S436/Z727 | 4 | 4202 | 5 |
| | single-copy | I1a1b3b | L813/S436/Z728 | 6 | 4202 | 8 |
| | single-copy | I1a1b3b | L813/S436/Z729 | 6 | 4202 | 9 |
| | single-copy | I1a1b3b | L813/S436/Z730 | 4 | 4194 | 4 |
| | single-copy | I1a1b3b | L813/S436/Z731 | 4 | 4202 | 4 |
| | single-copy | I1a1b3b | L813/S436/Z732 | 4 | 4202 | 5 |
| | single-copy | I1a1b3b | L813/S436/Z733 | 5 | 4202 | 7 |
| | single-copy | I1a1b3b | L813/S436/Z734 | 4 | 4202 | 7 |
| patriline 2 | single-copy | R1b1a1a2a1a1b1a | S375/Z372 | 4 | 4202 | 4 |
| | 2 copies, duplication | R1b1a1a2a1a1b1a | S375/Z373 | 4 | 4202 | 4 |
| | single-copy | R1b1a1a2a1a1b1a | S375/Z374 | 4 | 4202 | 5 |
| | 2 copies, no event | R1b1a1a2a1a1b1a | S375/Z375 | 56 | 4202 | 86 |
| | single-copy | R1b1a1a2a1a1b1a | S375/Z376 | 4 | 4202 | 5 |
| | single-copy | R1b1a1a2a1a1b1a | S375/Z377 | 5 | 4202 | 7 |
| patriline 3 | single-copy | I1a1b3b | L813/S436/Z734 | 4 | 4202 | 5 |
| | 2 copies, duplication | I1a1b3b | L813/S436/Z735 | 4 | 4202 | 4 |
| | single-copy | I1a1b3b | L813/S436/Z736 | 4 | 4201 | 2 |
| outgroup individuals | 2 copies, no event | I1a1b3b | L813/S436/Z737 | 56 | 4202 | 86 |
| | 2 copies, no event | I1a1b3b | L813/S436/Z738 | 53 | 4201 | 81 |
| | 2 copies, no event | R1b1a1a2a1a1b1a | S375/Z377 | 57 | 4197 | 89 |
| | 2 copies, no event | R1b1a1a2a1a1b1a | S375/Z378 | 55 | 4202 | 85 |

**Supplementary Table 15: Count and percentage of previously-reported deletions and duplications observed as the result of *de novo* events and in the phylogenetic tree**, and their respective mutation rate.

| | COUNTS (PERCENTAGE) | | | MUTATION RATE [95%CI] | |
|---|---|---|---|---|---|
| Event type | Patriline (MD) | Patriline (SD) | Tree | Patriline | Tree |
| b2/b4 del | 0 | 2 (4.4%) | 1 (1.2%) | 8.20E-05 [9.93E-06, 2.96E-04] | 1.19E-05 [6.22E-07, 7.74E-05] |
| b1/b3 del | 0 | 1 (2.2%) | 0 | 4.10E-05 [1.04E-06, 2.29E-04] | 0 |
| gr/gr del | 2 (11.8%) | 12 (26.6%) | 20 (24.1%) | 5.74E-04 [3.14E-04, 9.63E-04] | 2.38E-04 [1.50E-04, 3.75E-04] |
| gr/gr dup | 8 (47.1%) | 10 (22.2%) | 20 (24.1%) | 7.38E-04 [4.38E-04, 1.17E-03] | 2.38E-04 [1.50E-04, 3.75E-04] |
| b2/b4 dup | 2 (11.8%) | 2 (4.4%) | 2 (2.4%) | 1.64E-04 [4.47E-05, 4.20E-04] | 2.38E-05 [4.13E-06, 9.61E-05] |
| gr/gr del + b2/b4 dup | 1 (5.9%) | 2 (4.4%) | 0 | 1.23E-04 [2.54E-05, 3.60E-04] | 0 |
| b2/b3 del | 0 | 1 (2.2%) | 8 (9.6%) | 4.10E-05 [1.04E-06, 2.29E-04] | 9.53E-05 [4.43E-05, 1.96E-04] |
| b2/b3 or g1/g3 dup | 0 | 0 | 0 | 0 | 0 |

**Supplementary Table 16**: **Mutation probabilities** as calculated using the number of *de novo* events divided by the number of transmissions observed in the patrilines in each CN state for MD and SD events together. The mutation probabilities are also indicated for the 95% CI (proportion test). For example, the mutation probability from reference CN to non-reference CN is 0.0019.

| Values | mutation probabilities | | low 95% CI | | high 95% CI | |
|---|---|---|---|---|---|---|
| from/to | reference | non-reference | reference | non-reference | reference | non-reference |
| reference | 0.9981 | 0.0019 | 0.9975 | 0.0025 | 0.9986 | 0.0014 |
| non-reference | 0.0028 | 0.9972 | 0.0010 | 0.9990 | 0.0068 | 0.9932 |

**Supplementary Table 17: Grouping of the events detected in the Y chromosome phylogenetic tree in several amplicons.** GRY: green, red and yellow; RY: red and yellow; GRT: green, red and teal; GRYT: green, red, yellow and teal. The events are divided into multiple descendants (MD) and single descendants (SD) events; and into amplification (+) and deletion (-).

| type event | GRY | RY | GR | G | R | T | Y | Total |
|---|---|---|---|---|---|---|---|---|
| + | 23 | 1 | 4 | 5 | 1 | 1 | 0 | 35 |
| - | 22 | 1 | 0 | 1 | 0 | 0 | 1 | 25 |
| total | 45 | 2 | 4 | 6 | 1 | 1 | 1 | 60 |

**Supplementary Table 18: Comparison of the Z transformed mean number of children between individuals carrying the reference copy number and individuals carrying CNV**. The estimates and p-values are from single regression models, which corresponds to an ANOVA.

| | | Number of children | | Z transformed number of children | | | | | | |
|---|---|---|---|---|---|---|---|---|---|---|
| CN | N | Mean | sd | Z | sd | Median | min | max | Estimate | P-value |
| Reference | 10460 | 2.44 | 1.74 | 0.00 | 0.99 | -0.12 | -1.96 | 8.16 | - | - |
| Non-reference | 927 | 2.46 | 1.78 | 0.03 | 1.05 | 0.08 | -1.96 | 6.71 | 0.03 | 0.422 |
| Deletion | 613 | 2.44 | 1.71 | 0.00 | 1.04 | -0.12 | -1.96 | 6.71 | 0.00 | 0.976 |
| Duplication | 314 | 2.49 | 1.93 | 0.08 | 1.09 | 0.13 | -1.96 | 4.36 | 0.08 | 0.169 |
| gr/gr deletion | 495 | 2.42 | 1.69 | -0.01 | 1.05 | -0.12 | -1.96 | 6.71 | -0.01 | 0.778 |
| gr/gr deletion + b2/b4 duplication | 21 | 1.62 | 1.69 | -0.57 | 1.03 | -0.64 | -1.96 | 0.92 | -0.57 | 0.009 |
| b2/b3 or g1/g3 duplication | 20 | 3.35 | 1.93 | 0.69 | 0.88 | 1.02 | -0.84 | 1.71 | 0.69 | 0.002 |
| gr/gr duplication | 149 | 2.63 | 1.93 | 0.13 | 1.03 | 0.18 | -1.94 | 3.76 | 0.13 | 0.105 |
| b2/b3 deletion | 80 | 2.65 | 1.87 | 0.07 | 0.97 | 0.10 | -1.83 | 2.78 | 0.08 | 0.497 |
| b1/b3 deletion | 1 | 3.00 | NA | 0.40 | NA | 0.40 | 0.40 | 0.40 | 0.40 | 0.688 |
| b2/b4 duplication | 6 | 1.67 | 1.51 | -0.71 | 0.82 | -0.61 | -1.71 | 0.18 | -0.70 | 0.084 |
| AZFc | 2 | 2.00 | 2.83 | -0.26 | 2.11 | -0.26 | -1.75 | 1.23 | -0.26 | 0.711 |
| De novo (All) | 91 | 2.27 | 1.99 | 0.04 | 1.27 | 0.13 | -1.96 | 4.36 | 0.63 | 0.088 |
| De novo deletion | 42 | 2.02 | 1.65 | 0.00 | 1.34 | -0.20 | -1.96 | 4.36 | 0.40 | 0.333 |
| De novo duplication | 56 | 2.32 | 2.19 | -0.02 | 1.19 | 0.00 | -1.94 | 3.76 | 0.68 | 0.072 |

**Supplementary Table 19: Comparison of the proportion of individuals with no children between individuals carrying the reference copy number and individuals carrying CNV**, using a Fisher exact test (one sided, H0: proportion of individuals with 0 children is greater in the non-reference CN), for individuals born before or in 1982.

| | Proportion of individuals with 0 children (Fisher exact test) | | | | |
|---|---|---|---|---|---|
| CN | N | N0 | prop [95% CI] | p-value | odds ratio |
| Reference | 9027 | 961 | 0.11 [0.10, 0.11] | - | - |
| Non-reference | 805 | 96 | 0.12 [0.10, 0.14] | 0.26 | 1.13 |
| Deletion | 543 | 63 | 0.12 [0.09, 0.15] | 0.47 | 1.10 |
| Duplication | 262 | 33 | 0.13 [0.81, 1.76] | 0.31 | 1.21 |
| gr/gr deletion | 439 | 53 | 0.12 [0.09, 0.16] | 0.34 | 1.15 |
| gr/gr deletion + b2/b4 duplication | 19 | 6 | 0.32 [0.14, 0.57] | 0.01 | 3.87 |
| b2/b3 or g1/g3 duplication | 17 | 0 | 0.00 [0.00, 0.23] | 0.25 | 0.00 |
| b2/b3 deletion | 71 | 7 | 0.10 [0.04, 0.20] | 1.00 | 0.92 |
| gr/gr duplication | 128 | 13 | 0.10 [0.06, 0.17] | 1.00 | 0.95 |
| De novo (All) | 72 | 15 | 0.21 [0.12, 0.32] | 0.01 | 2.21 |
| De novo deletions | 31 | 7 | 0.23 [0.10, 0.42] | 0.04 | 2.45 |
| De novo duplications | 41 | 8 | 0.20 [0.09, 0.35] | 0.07 | 2.03 |

**Supplementary Table 20**: **Genomic position of the region used to calculate copy number from the median coverage** for each amplicon or palindromic arm. Includes the genomic positions and the relative positions within the palindromic arm included in the reference sequence constructed. To consider systematic low coverage in a region, some regions were separated in two for the calculation of the median coverage.

| palindrome | amplicon | start_pal | end_pal | start | end | relative_start | relative_end | length |
|---|---|---|---|---|---|---|---|---|
| palindrome1.proximal.arm | yellow | 23356858 | 24824727 | 23707857 | 23992775 | 351000 | 635918 | 284919 |
| palindrome1.proximal.arm | yellow2 | 23356858 | 24824727 | 24142857 | 24276001 | 786000 | 919144 | 133145 |
| palindrome1.proximal.arm | green | 23356858 | 24824727 | 24398041 | 24618004 | 1041184 | 1261147 | 219964 |
| palindrome1.proximal.arm | green2 | 23356858 | 24824727 | 24646857 | 24695498 | 1290000 | 1338641 | 48642 |
| palindrome1.proximal.arm | red | 23356858 | 24824727 | 24695499 | 24763069 | 1338642 | 1406212 | 67571 |
| palindrome3.proximal.arm | blue | 21904889 | 22208735 | 21925349 | 22064756 | 20461 | 159868 | 139408 |
| palindrome3.proximal.arm | teal1 | 21904889 | 22208735 | 22107007 | 22124965 | 202119 | 220077 | 17959 |
| palindrome3.proximal.arm | teal2 | 21904889 | 22208735 | 22150831 | 22165542 | 245943 | 260654 | 14712 |
| palindrome3.proximal.arm | teal3 | 21904889 | 22208735 | 22185942 | 22208738 | 281054 | 303850 | 22797 |
| palindrome4.proximal.arm | region1 | 18430214 | 18754061 | 18450000 | 18540000 | 19787 | 109787 | 90001 |
| palindrome4.proximal.arm | region2 | 18430214 | 18754061 | 18600000 | 18640000 | 169787 | 209787 | 40001 |
| palindrome5.proximal.arm | region1 | 17452347 | 17954719 | 17600000 | 17760000 | 147654 | 307654 | 160001 |
| palindrome5.proximal.arm | region2 | 17452347 | 17954719 | 17780000 | 17830000 | 327654 | 377654 | 50001 |
| palindrome6.proximal.arm | region1 | 16139395 | 16289395 | 16160000 | 16265000 | 20606 | 125606 | 105001 |
| palindrome7.proximal.arm | region1 | 15862230 | 15885378 | 15875000 | 15881000 | 12771 | 18771 | 6001 |
| palindrome8.proximal.arm | region1 | 13981005 | 14023073 | 13991000 | 14018000 | 9996 | 36996 | 27001 |

**Supplementary Table 21**: **Genomic position of the region used to calculate copy number from the HMM**, for each amplicon or palindromic arm. Includes the genomic positions and the relative positions within the palindromic arm included in the reference sequence constructed.

| palindrome | start | end | amplicon | relative start | relative end | start amplicon | end amplicon | length |
|---|---|---|---|---|---|---|---|---|
| palindrome1.proximal.arm | 23356858 | 24824727 | yellow | 351000 | 1000000 | 23707857 | 24356857 | 649001 |
| palindrome1.proximal.arm | 23356858 | 24824727 | green | 1041184 | 1338641 | 24398041 | 24695498 | 297458 |
| palindrome1.proximal.arm | 23356858 | 24824727 | red | 1339000 | 1405000 | 24695857 | 24761857 | 66001 |
| palindrome3.proximal.arm | 21904889 | 22208735 | blue | 20461 | 159868 | 21925349 | 22064756 | 139408 |
| palindrome3.proximal.arm | 21904889 | 22208735 | teal | 202119 | 303850 | 22107007 | 22208738 | 101732 |
| palindrome4.proximal.arm | 18430214 | 18754061 | NA | 19787 | 209787 | 18450000 | 18640000 | 190001 |
| palindrome5.proximal.arm | 17452347 | 17954719 | NA | 147654 | 377654 | 17600000 | 17830000 | 230001 |
| palindrome6.proximal.arm | 16139395 | 16289395 | NA | 20606 | 125606 | 16160000 | 16265000 | 105001 |
| palindrome7.proximal.arm | 15862230 | 15885378 | NA | 12771 | 18771 | 15875000 | 15881000 | 6001 |
| palindrome8.proximal.arm | 13981005 | 14023073 | NA | 9996 | 36996 | 13991000 | 14018000 | 27001 |

**Supplementary Table 22**: **Genomic position of the region removed to calculate copy number from the HMM**, for each amplicon or palindromic arm. To consider systematic low coverage in a region, some regions were not considered in the computation of the HMM.

| palindrome | start | end | amplicon | gene | relative start | relative end | start | end | length |
|---|---|---|---|---|---|---|---|---|---|
| palindrome1.proximal.arm | 23356858 | 24824727 | yellow | IR5.3 | 635918 | 870000 | 23992775 | 24226857 | 234083 |
| palindrome1.proximal.arm | 23356858 | 24824727 | green | BPY2 | 1250000 | 1290000 | 24606857 | 24646857 | 40001 |
| palindrome3.proximal.arm | 21904889 | 22208735 | teal | TTTY6 | 220075 | 245941 | 22124963 | 22150829 | 25867 |
| palindrome3.proximal.arm | 21904889 | 22208735 | teal | RBMY1F | 260651 | 281051 | 22165539 | 22185939 | 20401 |
| palindrome4.proximal.arm | 18430214 | 18754061 | NA | NA | 109787 | 169787 | 18540000 | 18600000 | 60001 |
| palindrome5.proximal.arm | 17452347 | 17954719 | NA | NA | 307654 | 327654 | 17760000 | 17780000 | 20001 |

**Supplementary Table 23: Difference in median coverage between duplicates for each amplicon and palindromic arm**, with the variance and standard deviation of the difference.

| Amplicon | type | median | variance | sd |
|---|---|---|---|---|
| green | same sample | 0.045 | 0.004 | 0.061 |
| green | different sample | 0.117 | 0.017 | 0.132 |
| green | all | 0.111 | 0.016 | 0.126 |
| yellow | same sample | 0.023 | 0.001 | 0.032 |
| yellow | different sample | 0.083 | 0.006 | 0.076 |
| yellow | all | 0.071 | 0.005 | 0.072 |
| red | same sample | 0.034 | 0.008 | 0.089 |
| red | different sample | 0.150 | 0.023 | 0.153 |
| red | all | 0.143 | 0.022 | 0.149 |
| teal | same sample | 0.038 | 0.002 | 0.041 |
| teal | different sample | 0.091 | 0.009 | 0.096 |
| teal | all | 0.077 | 0.008 | 0.092 |
| P8 | same sample | 0.063 | 0.003 | 0.050 |
| P8 | different sample | 0.091 | 0.007 | 0.085 |
| P8 | all | 0.083 | 0.006 | 0.080 |
| P6 | same sample | 0.052 | 0.002 | 0.048 |
| P6 | different sample | 0.100 | 0.011 | 0.107 |
| P6 | all | 0.087 | 0.010 | 0.102 |
| P7 | same sample | 0.119 | 0.007 | 0.087 |
| P7 | different sample | 0.200 | 0.026 | 0.160 |
| P7 | all | 0.183 | 0.024 | 0.156 |
| P4 | same sample | 0.027 | 0.001 | 0.036 |
| P4 | different sample | 0.100 | 0.006 | 0.078 |
| P4 | all | 0.091 | 0.006 | 0.078 |
| P5 | same sample | 0.030 | 0.001 | 0.036 |
| P5 | different sample | 0.091 | 0.006 | 0.079 |
| P5 | all | 0.083 | 0.006 | 0.076 |

**Supplementary Table 24: Count of the individuals with a different CN between duplicates as estimated by the HMM**, for different sample type and same sample type. The false positive rate for each amplicon and palindromic arms is calculated on the right.

| | different sample type | | | | same sample type | | | | false positive rate | |
|---|---|---|---|---|---|---|---|---|---|---|
| Difference | -2 | -1 | 0 | 1 | -2 | -1 | 0 | 1 | different sample | total |
| green | 0 | 1 | 75 | 0 | 0 | 0 | 14 | 0 | 0.013 | 0.011 |
| red | 0 | 1 | 75 | 1 | 0 | 0 | 14 | 0 | 0.026 | 0.022 |
| teal | 0 | 0 | 77 | 0 | 0 | 0 | 14 | 0 | 0.000 | 0.000 |
| yellow | 0 | 0 | 77 | 0 | 0 | 0 | 14 | 0 | 0.000 | 0.000 |
| P4 | 0 | 0 | 77 | 0 | 0 | 0 | 14 | 0 | 0.000 | 0.000 |
| P5 | 0 | 0 | 77 | 0 | 0 | 0 | 14 | 0 | 0.000 | 0.000 |
| P6 | 0 | 0 | 77 | 0 | 0 | 0 | 14 | 0 | 0.000 | 0.000 |
| P7 | 0 | 0 | 77 | 2 | 0 | 0 | 14 | 0 | 0.024 | 0.021 |
| P8 | 0 | 0 | 77 | 0 | 0 | 0 | 14 | 0 | 0.000 | 0.000 |

**Supplementary Table 25: X-degenerate hg38 coordinates** used in calculating the average sequence depth per individual BAM file and in building the phylogenetic tree of the Y chromosome in the Icelandic population.

| Region | start | stop | length |
|---|---|---|---|
| XDG1 | 2781766 | 3049682 | 267917 |
| XDG2 | 6748711 | 7604183 | 855473 |
| XDG3 | 11749732 | 13983906 | 2234175 |
| XDG4 | 14058734 | 15874593 | 1815860 |
| XDG5 | 15905215 | 16159393 | 254179 |
| XDG6 | 16425966 | 17455476 | 1029511 |
| XDG7 | 18870335 | 20054272 | 1183938 |
| XDG8 | 20351234 | 21335775 | 984542 |
| XDG9 | 26311846 | 26660611 | 348766 |
| totals | | | 8974361 |

**Supplementary Table 26: Mean difference between the observed and the simulated proportions of individuals with non-reference CN**, for the selection coefficients (s).

| s | mean | sd |
|---|---|---|
| 0 | -0.199 | 0.062 |
| 0.004 | -0.121 | 0.032 |
| 0.008 | -0.071 | 0.019 |
| 0.012 | -0.033 | 0.007 |
| 0.014 | -0.025 | 0.006 |
| 0.016 | -0.011 | 0.006 |
| 0.018 | -0.002 | 0.003 |
| 0.02 | 0.005 | 0.003 |
| 0.022 | 0.009 | 0.002 |
| 0.024 | 0.017 | 0.003 |
| 0.026 | 0.021 | 0.002 |
| 0.03 | 0.028 | 0.002 |

## Supplementary References


1. Navarro-Costa, P., Gonçalves, J. & Plancha, C. E. The AZFc region of the Y chromosome: At the crossroads between genetic diversity and male infertility. *Hum. Reprod. Update* **16**, 525–542 (2010).
2. Jagadeesan, A. *et al.* HaploGrouper: A generalized approach to haplogroup classification. *Bioinformatics* **37**, 570–572 (2020).
3. Lucotte, E. A. *et al.* Dynamic Copy Number Evolution of X- and Y-Linked Ampliconic Genes in Human Populations. *Genetics* **209**, genetics.300826.2018 (2018).
4. Skov, L., The Danish Pan Genome Consortium & Schierup, M. H. Analysis of 62 hybrid assembled human Y chromosomes exposes rapid structural changes and high rates of gene conversion. *PLOS Genet.* **13**, e1006834 (2017).
5. Li, H. & Durbin, R. Fast and accurate short read alignment with Burrows-Wheeler transform. *Bioinformatics* **25**, 1754–1760 (2009).
6. Tarasov, A., Vilella, A. J., Cuppen, E., Nijman, I. J. & Prins, P. Sambamba: Fast processing of NGS alignment formats. *Bioinformatics* **31**, 2032–2034 (2015).
7. Li, H. *et al.* The Sequence Alignment/Map format and SAMtools. *Bioinformatics* **25**, 2078–2079 (2009).
8. Gudbjartsson, D. F. *et al.* Large-scale whole-genome sequencing of the Icelandic population. *Nat. Genet.* **47**, 435–444 (2015).
9. Harris, B., Riemer, C. & Miller, W. Lastz. (2010).
10. Lê, S., Josse, J. & Husson, F. {FactoMineR}: A Package for Multivariate Analysis. *J. Stat. Softw.* **25**, 1–18 (2008).
11. Hahsler, M., Piekenbrock, M. & Doran, D. {dbscan}: Fast Density-Based Clustering with {R}. *J. Stat. Softw.* **91**, 1–30 (2019).
12. Helgason, A. *et al.* The Y-chromosome point mutation rate in humans. *Nat. Genet.* **47**, 453–457 (2015).
13. Spedicato, G. A. Discrete Time Markov Chains with R. *R J.* (2017).
14. Simonsen, M., Mailund, T. & Pedersen, C. N. S. Rapid Neighbour-Joining. in *Algorithms in Bioinformatics* 113–122 (2008).
