# Supplementary material for: Characterizing the evolution and phenotypic impact of ampliconic Y chromosome regions
Source: Nat Commun. 2023 Jul 6;14:3990. doi: 10.1038/s41467-023-39644-6 (PMC10326017; doi:10.1038/s41467-023-39644-6)
Supplement: Supplementary file 3 — Description of Additional Supplementary Files [file 41467_2023_39644_MOESM3_ESM.pdf]

## Description of Additional Supplementary Files

**File Name**: Supplementary Data 1
**Description**: List of the pseudo-heterozygous positions used in calculating individual MARF values. Listing, per position, the hg38 coordinates of the pseudo-heterozygous positions and their paralogs, along with the reference allele and alternative allele(s) for the proximal arm, the number of individuals with valid genotypes and finally the number of individuals with pseudo-heterozygosity.

**File Name**: Supplementary Data 2
**Description**: Phenotypic association study comparing individuals with the reference CN and individuals with deletions or duplication. The phenotype type (binary or quantitative), the number of individuals measured for this phenotype, the value of the effect (beta) and the p-value of the test are indicated. Logistic regressions were performed for binary phenotypes, and linear regression were performed for quantitative phenotypes. After correction for multiple testing (Bonferroni), no p-value is significant.

**File Name**: Supplementary Data 3
**Description**: Phenotypic association study comparing individuals with the reference CN and individuals with deletions. The phenotype type (binary or quantitative), the number of individuals measured for this phenotype, the value of the effect (beta) and the p-value of the test are indicated. Logistic regressions were performed for binary phenotypes, and linear regression were performed for quantitative phenotypes After correction for multiple testing (Bonferroni), no p-value is significant.

**File Name**: Supplementary Data 4
**Description**: Phenotypic association study comparing individuals with the reference CN and individuals with duplication. The phenotype type (binary or quantitative), the number of individuals measured for this phenotype, the value of the effect (beta) and the p-value of the test are indicated. Logistic regressions were performed for binary phenotypes, and linear regression were performed for quantitative phenotypes. After correction for multiple testing (Bonferroni), no p-value is significant.
