# Supplementary material for: Characterizing the evolution and phenotypic impact of ampliconic Y chromosome regions
Source: Nat Commun. 2023 Jul 6;14:3990. doi: 10.1038/s41467-023-39644-6 (PMC10326017; doi:10.1038/s41467-023-39644-6)
Supplement: Supplementary file 8 — Reporting Summary [file 41467_2023_39644_MOESM8_ESM.pdf]

nature portfolio

Corresponding author(s): Elise Anne Lucotte

Last updated by author(s): Mar 10, 2023

Reporting Summary

Nature Portfolio wishes to improve the reproducibility of the work that we publish. This form provides structure for consistency and transparency in reporting. For further information on Nature Portfolio policies, see our Editorial Policies and the Editorial Policy Checklist.

Statistics

For all statistical analyses, confirm that the following items are present in the figure legend, table legend, main text, or Methods section.

n/a	Confirmed	
☐	☒	The exact sample size (*n*) for each experimental group/condition, given as a discrete number and unit of measurement
☐	☒	A statement on whether measurements were taken from distinct samples or whether the same sample was measured repeatedly
☐	☒	The statistical test(s) used AND whether they are one- or two-sided<br>*Only common tests should be described solely by name; describe more complex techniques in the Methods section.*
☐	☒	A description of all covariates tested
☐	☒	A description of any assumptions or corrections, such as tests of normality and adjustment for multiple comparisons
☐	☒	A full description of the statistical parameters including central tendency (e.g. means) or other basic estimates (e.g. regression coefficient) AND variation (e.g. standard deviation) or associated estimates of uncertainty (e.g. confidence intervals)
☐	☒	For null hypothesis testing, the test statistic (e.g. *F*, *t*, *r*) with confidence intervals, effect sizes, degrees of freedom and *P* value noted<br>*Give P values as exact values whenever suitable.*
☒	☐	For Bayesian analysis, information on the choice of priors and Markov chain Monte Carlo settings
☒	☐	For hierarchical and complex designs, identification of the appropriate level for tests and full reporting of outcomes
☐	☒	Estimates of effect sizes (e.g. Cohen's *d*, Pearson's *r*), indicating how they were calculated

Our web collection on statistics for biologists contains articles on many of the points above.

Software and code

Policy information about availability of computer code

Data collection	The description of the data acquisition and processing is described in the Data Descriptor (Jónsson, H. et al. Whole genome characterization of sequence diversity of 15,220 Icelanders. Sci. Data 4:170115 doi: 10.1038/sdata.2017.115 (2017).)
Data analysis	Custom scripts written in python 2.7 and python 3<br>BWA v0.7.5<br>sambamba v0.5.1<br>SAMtools v1.3<br>R v3.6.1

For manuscripts utilizing custom algorithms or software that are central to the research but not yet described in published literature, software must be made available to editors and reviewers. We strongly encourage code deposition in a community repository (e.g. GitHub). See the Nature Portfolio guidelines for submitting code & software for further information.

Data

Policy information about availability of data

All manuscripts must include a data availability statement. This statement should provide the following information, where applicable:

- Accession codes, unique identifiers, or web links for publicly available datasets
- A description of any restrictions on data availability
- For clinical datasets or third party data, please ensure that the statement adheres to our policy

Icelandic law and the regulations of the Icelandic Data Protection Authority prohibit the release of individual level and personally identifying data. We are actively participating in multiple meta-analysis based on our data and are in collaboration with groups at over 100 international universities and institutions. Therefore, collaborations based on our sequencing data are based on the release of summary level statistics, such as effect sizes and P-values for meta-analysis, or the collaborators travelling to our Icelandic facilities for local data access. Contact the corresponding author Agnar Helgason (agnar.helgason@decode.is) for more details.

Human research participants

Policy information about studies involving human research participants and Sex and Gender in Research.

Reporting on sex and gender	The study is on Y chromosome, therefore only individuals with the sex male attributed at birth were considered.
Population characteristics	All 11525 samples come from Icelandic men, whole genome sequenced using PCR-free methods. The samples were collected and sequenced by deCODE Genetics. When available, the number of children of each individual was used as a fertility measurement.
Recruitment	Recruitment is based on volunteers providing samples for particular disease and/or control studies either directly or through clinical collaborators. We are not aware of any recruitment biases that would impact the results of this study
Ethics oversight	The study is undertaken on the basis of approvals from the National Bioethics Committee and the Icelandic Data Protection Authority. Blood or buccal samples were taken from individuals participating in various studies, after receiving informed consent from them or their guardians.

Note that full information on the approval of the study protocol must also be provided in the manuscript.

Field-specific reporting

Please select the one below that is the best fit for your research. If you are not sure, read the appropriate sections before making your selection.

☒ Life sciences ☐ Behavioural & social sciences ☐ Ecological, evolutionary & environmental sciences

For a reference copy of the document with all sections, see nature.com/documents/nr-reporting-summary-flat.pdf

Life sciences study design

All studies must disclose on these points even when the disclosure is negative.

Sample size	11527 males, the largest sample of the kind to date.
Data exclusions	All 11527 males were included in the copy number assessment analyses. Then, only individuals from patrilines were included to estimate the mutation rate (N=7947)
Replication	Copy number genotypes were corroborated with two different analyses using an independent mapping of the reads than the one used for copy number genotype calls: 1) calculation of read frequencies at paralogous positions with allelic variation and 2) of read depth. Moreover, to assess the false positive rate of copy number variation calling within patrilines, we included five additional men with WGS data who were not included in the original analyses and who are descendants of lineages with inferred de novo events. The results were therefore replicated completely once, and partially once. The findings were replicated both times.
Randomization	Randomization is not relevant to our study because we performed population genetics analyses.
Blinding	The investigators were not blinded to allocation during experiments and outcome assessment. The same persons worked on determining Y chromosome copy number and the rest of the analyses.

Reporting for specific materials, systems and methods

We require information from authors about some types of materials, experimental systems and methods used in many studies. Here, indicate whether each material, system or method listed is relevant to your study. If you are not sure if a list item applies to your research, read the appropriate section before selecting a response.

Materials & experimental systems

n/a	Involved in the study
☒	☐ Antibodies
☒	☐ Eukaryotic cell lines
☒	☐ Palaeontology and archaeology
☒	☐ Animals and other organisms
☒	☐ Clinical data
☒	☐ Dual use research of concern

Methods

n/a	Involved in the study
☒	☐ ChIP-seq
☒	☐ Flow cytometry
☒	☐ MRI-based neuroimaging
